# The evolutionary and genetic origins of consciousness in the Cambrian Period over 500 million years ago

**DOI:** 10.3389/fpsyg.2013.00667

**Published:** 2013-10-04

**Authors:** Todd E. Feinberg, Jon Mallatt

**Affiliations:** ^1^Neurology and Psychiatry, Albert Einstein College of Medicine and Beth Israel Medical CenterNew York, NY, USA; ^2^School of Biological Sciences, Washington State UniversityPullman, WA, USA

**Keywords:** isomorphic/somatotopic neural representations, sensory images, qualia, neural crest and placodes, lamprey, genes of consciousness, optic tectum and consciousness, thalamocortical complex

## Abstract

Vertebrates evolved in the Cambrian Period before 520 million years ago, but we do not know when or how consciousness arose in the history of the vertebrate brain. Here we propose multiple levels of *isomorphic or somatotopic neural representations* as an objective marker for sensory consciousness. All extant vertebrates have these, so we deduce that consciousness extends back to the group's origin. The first conscious sense may have been vision. Then vision, coupled with additional sensory systems derived from ectodermal placodes and neural crest, transformed primitive reflexive systems into *image forming brains* that map and perceive the external world and the body's interior. We posit that the minimum requirement for sensory consciousness and *qualia* is a brain including a forebrain (but not necessarily a developed cerebral cortex/pallium), midbrain, and hindbrain. This brain must also have (1) hierarchical systems of intercommunicating, isomorphically organized, processing nuclei that extensively integrate the different senses into representations that emerge in upper levels of the neural hierarchy; and (2) a widespread reticular formation that integrates the sensory inputs and contributes to attention, awareness, and neural synchronization. We propose a two-step evolutionary history, in which the optic tectum was the original center of multi-sensory conscious perception (as in fish and amphibians: step 1), followed by a gradual shift of this center to the dorsal pallium or its cerebral cortex (in mammals, reptiles, birds: step 2). We address objections to the hypothesis and call for more studies of fish and amphibians. In our view, the lamprey has all the neural requisites and is likely the simplest extant vertebrate with sensory consciousness and qualia. Genes that pattern the proposed elements of consciousness (isomorphism, neural crest, placodes) have been identified in all vertebrates. Thus, consciousness is in the genes, some of which are already known.

## Introduction

But no matter how the form may vary, the fact that an organism has conscious experience at all means, basically, that there is something it is like to be that organism. …fundamentally an organism has conscious mental states if and only if there is something that it is like to *be* that organism - something it is like *for* the organism. We may call this the subjective character of experience (Nagel, 1974, p. 436).

Although there are many aspects to the nature of consciousness, this paper focuses on the neurological basis and evolutionary origins of *sensory consciousness*. Sensory consciousness is akin to concepts such as *phenomenal consciousness* (Revonsuo, 2006, 2010; Boly and Seth, 2012), *primary consciousness* (Edelman, 1989), *subjectivity* (Nagel, 1989; Searle, 1992, 1997; Tye, 2000; Metzinger, 2003; Velmans, 2009; Feinberg, 2012), or the experience of *qualia* (Churchland and Churchland, 1981; Jackson, 1982; Levine, 1983; Dennett, 1988, 1991; Flanagan, 1992; Kirk, 1994; Chalmers, 1995, 1996, 2010; McGinn, 1999; Metzinger, 2003). Other studies explore the evolutionary origin of consciousness in memory and learning, for goal-directed actions and behaviors, or in arousal and emotions (Ginsburg and Jablonka, 2010, 2011; Mashour and Alkire, 2013), but again, our focus will be on sensory experience.

This is because the subjective nature of qualia is so important. Chalmers sees it as the central issue of the problem of consciousness:

If any problem qualifies as *the* problem of consciousness, it is this one. In this central sense of “consciousness”, an organism is conscious if there is something it is like to be that organism, and a mental state is conscious if there is something it is like to be in that state. Sometimes terms such as “phenomenal consciousness” and “qualia” are also used here, but I find it more natural to speak of “conscious experience” or simply “experience” (Chalmers, 1995, p. 201).

The current inability to understand such experiences is called the explanatory gap (see Block, 2009), and Crick and Koch agree that the puzzle of sensory subjectivity must be solved for progress to be made:

The most difficult aspect of consciousness is the so-called ‘hard problem’ of qualia,—the redness of red, the painfulness of pain, and so on. No one has produced any plausible explanation as to how the experience of the redness of red could arise from the actions of the brain (Crick and Koch, 2003, p. 119).

Here we take on this fundamental problem from the non-traditional perspective of evolutionary, developmental, and genetic neurobiology.

Revonsuo's definition of phenomenal consciousness expands the description of the subjective, phenomenal, and sensory aspects of consciousness:

Phenomenal consciousness is the current *presence* of subjective experiences, or the *having* of subjective experiences. An organism possesses phenomenal consciousness if there is any type of subjective experience currently present for it. The mere occurrence or presence of any experience is the necessary and minimally sufficient condition for phenomenal consciousness. For any entity to possess primary phenomenal consciousness only requires that there are at least *some* patterns – any patterns at all – of subjective experience *present-for-it*. It is purely about the *having* of *any* sorts of patterns of subjective experience, whether simple or complex, faint or vivid, meaningful or meaningless, fleeting or lingering (Revonsuo, 2006, p. 37).

In this paper, when we refer to “sensory consciousness” we are referring to these unique, phenomenal, subjective features of consciousness (Feinberg, 2009, 2011, 2012). But if we are to deepen our understanding of the evolutionary origins and neurobiological basis of sensory consciousness, we must first face the difficult task of translating descriptions of neurological structure and function into concepts that describe subjective experience.

What organisms are likely to possess phenomenal consciousness or subjective states of awareness? Clearly, whether one deems an animal “conscious” depends upon what criteria are employed (Cartmill, 2000; Griffin, 2000; Butler et al., 2005; Edelman et al., 2005; Seth et al., 2005; Edelman and Seth, 2009). The question of consciousness has been studied in intellectually advanced, large-brained animals such as non-human primates, birds, octopuses, and squids (e.g., Pennisi, 1999; Butler et al., 2005; Edelman et al., 2005; Seth et al., 2005; Butler, 2008a; Mather, 2008; Edelman and Seth, 2009), but we wish to explicate the simplest neural architecture most relevant to human consciousness and therefore will focus on the earliest appearance of sensory consciousness in vertebrate-craniate evolution (Northcutt, 1996a,b; Hodos and Butler, 1997; Nieuwenhuys and Nicholson, 1998; Butler, 2000; Holland and Holland, 2001; Butler and Hodos, 2005; Lacalli, 2008a,b; Fritzsch and Glover, 2009; Glover and Fritzsch, 2009; Kaas, 2009). Because usage of the names “vertebrate” vs. “craniate” is confused and in flux (Kardong, 2012), we will keep things simple by using both names synonymously to designate the group of animals with a vertebral column and skull. These are the fish, amphibians, reptiles, birds, and mammals of common parlance.

While no single concept or approach to sensory consciousness can subsume all others, we take as a starting point the question of how an essentially neurological concept—the concept of “somatotopic,” “topographic,” and “isomorphic” sensory maps or representations—can be translated into simple ideas or terms that have a clear meaning from the standpoint of subjective awareness, without getting too deeply entrenched in the many complex and thorny philosophical issues that this approach might entail.

“*Isomorphic map”* (Hodos and Butler, 1997) is a general term for neural representations that are organized spatially according to different points in the sensory field or in the outside world being sensed (retinotopic, somatotopic, nociceptive, or cochleotopic and thereby tonotopic) as well as the non-spatially organized, chemotopically mapped representations (olfactory and gustatory) (Barlow, 1981, 1986; Northcutt and Kaas, 1995; Kaas, 1997; Leon and Johnson, 2003; Shepherd, 2007; Thivierge and Marcus, 2007; Gottfried, 2010). It is generally held that isomorphic maps are essential to sensory functioning in vertebrates: These maps persist through a hierarchy of successive and interconnected processing stations, with the topographical organization becoming progressively more complex in the higher stations in the brain (Kaas, 1997). Here we propose that certain sorts of complex, integrated isomorphic representations are associated with conscious scenes, and the purpose of this paper is to explore the implications of this assumption across a larger range of vertebrate animals, and in considerably more biological detail, than has been done previously.

Although the isomorphic map is a fundamental and shared trait, the different sensory systems have some special features and variations in their maps. For example, while the somatotopic maps for “touch” roughly preserve the spatial relationship between their respective receptor surfaces and their central neural representations, some of these maps, such as in the somatosensory homunculus within SI of the postcentral gyrus of the mammalian cerebral cortex, are in reality splits or gross distortions of the body surface, reflecting additional features such as the greater density of peripheral innervation in some body regions (Kaas, 1997; Merker, 2007). Another map, in the vestibular cortex and involved with the sense of equilibrium, is both genuinely somatotopic (Grusser et al., 1990; Lopez and Blanke, 2011) and “directionally isomorphic,” consciously sensing movements of the body through 3D space (Chen et al., 2010). The chemotopic maps of olfactory and gustatory functioning are spatial at only some levels of their sensory pathways and not spatially organized at other levels (Sewards and Sewards, 2001; Rawson and Yee, 2006; Hara, 2007; Sosulski et al., 2011; Jacobs, 2012), so it is best to refer to these maps as isomorphic alone, still representing a hierarchical neural mapping for the construction of a sensory image (i.e., of different odorants or tastants).

We propose that, when considered from the point of view of the conscious human or non-human animal, the high-order isomorphic neural-representations are experienced as *sensory mental images*. We use the term “sensory mental image” to describe those aspects of phenomenal consciousness that are the direct and immediate result of the brain's processing of sensory information, much the same way as Gerald Edelman defines *primary consciousness* as “the state of being mentally aware of things in the world—of having mental images in the present” (Edelman, 1992). Other studies have also suggested that isomorphic maps are critical to the creation of sensory consciousness (Edelman, 1989; Damasio, 1999; Feinberg, 2009, 2011, 2012). Note that this use of the term “mental image” is not the same as “mental imagery” that results from mental imagining in the absence of an immediate stimulus. By our reasoning, an organism with a nervous system that translates its sensory arrays into mental images through central processing (see Table 1) possesses at least a minimal form of sensory, phenomenal, or primary consciousness. Note from the table that conscious images would only emerge with contributions from the third- and higher orders in mammals (cerebral cortex), and the second order in fishes and amphibians (optic tectum).

**Table 1:** **A simplified summary of some of the major sensory receptors and isomorphic pathways leading to sensory mental images**.

**Isomorphic templates**			
**Sensory domain, receptor type**	**First order multipolar**	**Second order**	Third order^*^ isomorphism, image type
Vision, photoreceptors: rods and cones	Retina = retinal ganglion cells	Thalamus = lateral geniculate, optic tectum^*^	Primary visual cortex (V1), retinotopic, *visual images*
Somesthetic senses, mechanoreceptors	Dorsal column nuclei (trunk), sensory trigeminal nerve nuclei (face)	Thalamus = VPL and VPM, tectum^*^	Primary somatosensory cortex (SI), somatotopic, *somatosensory images*
Pain, nociceptors	Dorsal horn lamina I (trunk), sensory trigeminal nuclei (face)	Thalamus = VPL/VPM, VMpo, tectum^*^	SI and insula-anterior cingulate, somatotopic-homeostatic, *pain images*
Olfaction, chemoreceptors: olfactory sensory neurons	Olfactory bulb = glomeruli: mitral cells	Olfactory cortex	Orbitofrontal cortex, chemotopic, *olfactory images*.
			Hippocampus and dentate gyrus, *olfactory images*
Gustation, chemoreceptors: taste cells	Gustatory nucleus	Thalamus = VPMpc, tectum^*^	Anterior insula/frontal operculum, chemotopic, *taste images*
Audition, mechanoreceptors: inner hair cells	Cochlear nuclei	Thalamus = medial geniculate, inferior colliculus, tectum^*^	Primary auditory, cortex, tonotopic, *auditory images*
Equilibrium, mechanoreceptors: hair cells	Vestibular nuclei	Thalamus = multiple thalamic nuclei, tectum^*^	Primary vestibular cortex (parieto-insular vestibular cortex: PIVC), *images of body position and motion*

* *Here the third-order telencephalic areas are listed for mammals, but higher levels also exist: Heteromodal association cortices (also designated as high-order association cortex, polymodal cortex, multimodal cortex, polysensory areas, and supramodal cortex) serve as fourth-order integration zones, and in the human brain they include the posterior and anterior parietal cortex, lateral temporal cortex, prefrontal cortex, and portions of the parahippocampal gyrus (Mesulam, 2000). In birds, the third and fourth orders also are in the pallium (Butler et al., 2005, 2011). In fish and amphibians, by contrast, the optic tectum is where the isomorphic visual, somatosensory, auditory, vestibular and nociceptive templates and images are best documented, as are the multimodal images (Echteler, 1985; McHaffie et al., 1989; Stein and Meredith, 1993; Merker, 2005, 2007; Dicke and Roth, 2009; Wullimann and Vernier, 2009a)*.

In this paper, we deduce that consciousness evolved in the earliest vertebrates in the Cambrian, the oldest geologic period with abundant fossil evidence for complex animals (Erwin and Valentine, 2013). A few other authors have also proposed that changes at this time were associated with the origin of consciousness. Hameroff (1998) suggested that consciousness first evolved in the Cambrian in simple worms, urchins, or even in one-celled organisms (suctorians), due to quantum effects at the level of microtubules in their cells. Ginsburg and Jablonka (2010, 2011) argued for an even earlier origin of consciousness than we do, in the pre-Cambrian, Ediacaran Period with the very first appearance of worm-like bilaterian animals; and that this consciousness coincided with the evolution of associative learning and memory. With reference to Hameroff (1998) and Ginsburg and Jablonka (2010, 2011), we will argue that simple bilaterians or one-celled creatures are not conscious because consciousness requires a more-complex nervous system. Additionally, with reference to Ginsburg and Jablonka we argue that learning and memory are well documented in simple animals like worms and *Aplysia* (snail-like sea slug: Hawkins et al., 2006; Kandel, 2009), yet according to our hypothesis they are not conscious. In fact, even computers have memory and can be programmed to learn without consciousness.

We avoid the intricacies of cognition- and quantum-based approaches to consciousness by focusing on sensory experience, which seems to be a more solvable problem in terms of current neurobiological knowledge. In fact, our tactic of analyzing the basis and origins of isomorphic sensory images has the unique advantage of *triangulating* between the neurobiological domain (of somatotopy, isomorphism, neurohierarchical pathways), neuropsychological domain (of sensory images) and neurophilosophical domain (of subjective experience and the hard problem) and thus could serve as a versatile analytic tool with wide application to these various approaches. Using multi-level, isomorphic sensory representations as an objective “marker” for the presence of sensory consciousness, we will consider: *How and when did isomorphic sensory images evolve? And what are the simplest extant vertebrates that have them?* Finally, we will consider the implications of this analysis for a neuroscience and a genetics of consciousness.

## The birth of brains

**Figure 1:**
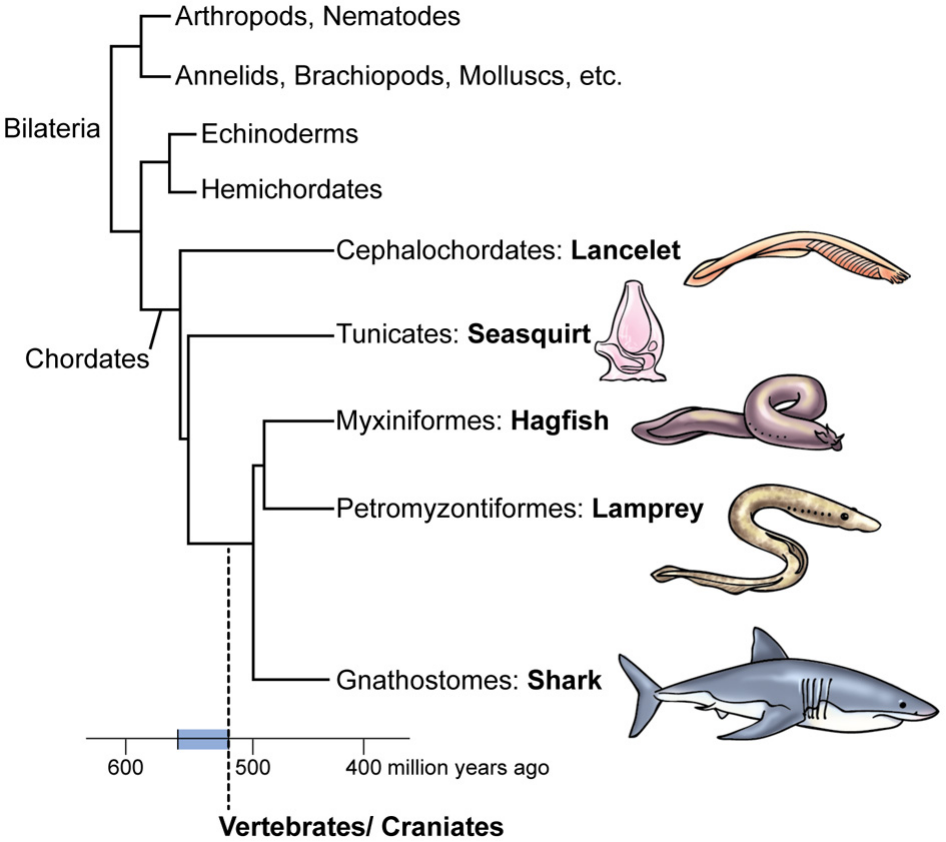
**Tree and timeline of the evolution of vertebrates**. Note the subgroups of the chordates. The vertebrate/craniate lineage evolved during the Cambrian Period approximately 560–520 million years ago (blue bar on the timeline). The two basic subdivisions of vertebrates are the jawless cyclostomes, namely hagfish and lamprey, and the jawed gnathostomes, to which we humans belong. The lamprey is thought to retain more features of the ancestral first vertebrate than do hagfish or gnathostomes.

Members of the phylum Chordata, including humans, are characterized by the presence at some point in their life cycle of a notochord (an elongated cellular chord that provides structural support for the animal's body) and a dorsal nerve cord. The chordates consist of three subphyla: Vertebrata, Cephalochordata, and Urochordata or tunicates (Figure 1) (Kardong, 2012). The vertebrates include the jawless hagfish and lampreys (cyclostomes), as well as the jawed vertebrates (gnathostomes). The gnathostomes consist of the jawed fish (cartilaginous and bony fish), amphibians, reptiles, birds, and mammals. The reptiles, birds, and mammals comprise the amniotes, so all the other vertebrates are called anamniotes (fish and amphibians). The first amniotes appeared in the Late Paleozoic Era about 350–330 mya, and fossils reveal they were extremely reptile-like, even lizard-like in appearance. Later, about 315 mya, the amniotes split into two lines: the synapsids (mammal-like reptiles and later, their mammal descendants); and the sauropsids, which include the living reptiles and the birds (“feathered dinosaurs”) (Kemp, 1982; Benton and Donoghue, 2007; Organ et al., 2007). Actually, the position of turtles among reptiles is uncertain because some evidence places turtles in sauropsids and other evidence indicates they arose before the sauropsid/synapsid split (Mallatt and Winchell, 2007).

Turning to the non-vertebrate chordates, which are informally called protochordates, the tunicate urochordates (also known as sea squirts) have a free-swimming larval phase during which they possess the chordate-defining notochord and nerve cord, and a bag-like adult form that is sessile (non-mobile) and anchored in one place on the ocean bottom (Burighel and Cloney, 1997). The cephalochordates (lancelets or amphioxus) are 4–6 cm long fish-shaped animals in which both the larvae and adults swim well and whose notochord and nerve cord persist their entire lives (Ruppert, 1997; Nieuwenhuys and Nicholson, 1998; Allman, 1999; Butler, 2000; Butler and Hodos, 2005; Fritzsch and Glover, 2009; Glover and Fritzsch, 2009; Kardong, 2012). The adults live burrowed in ocean sediment.

**Figure 2:**
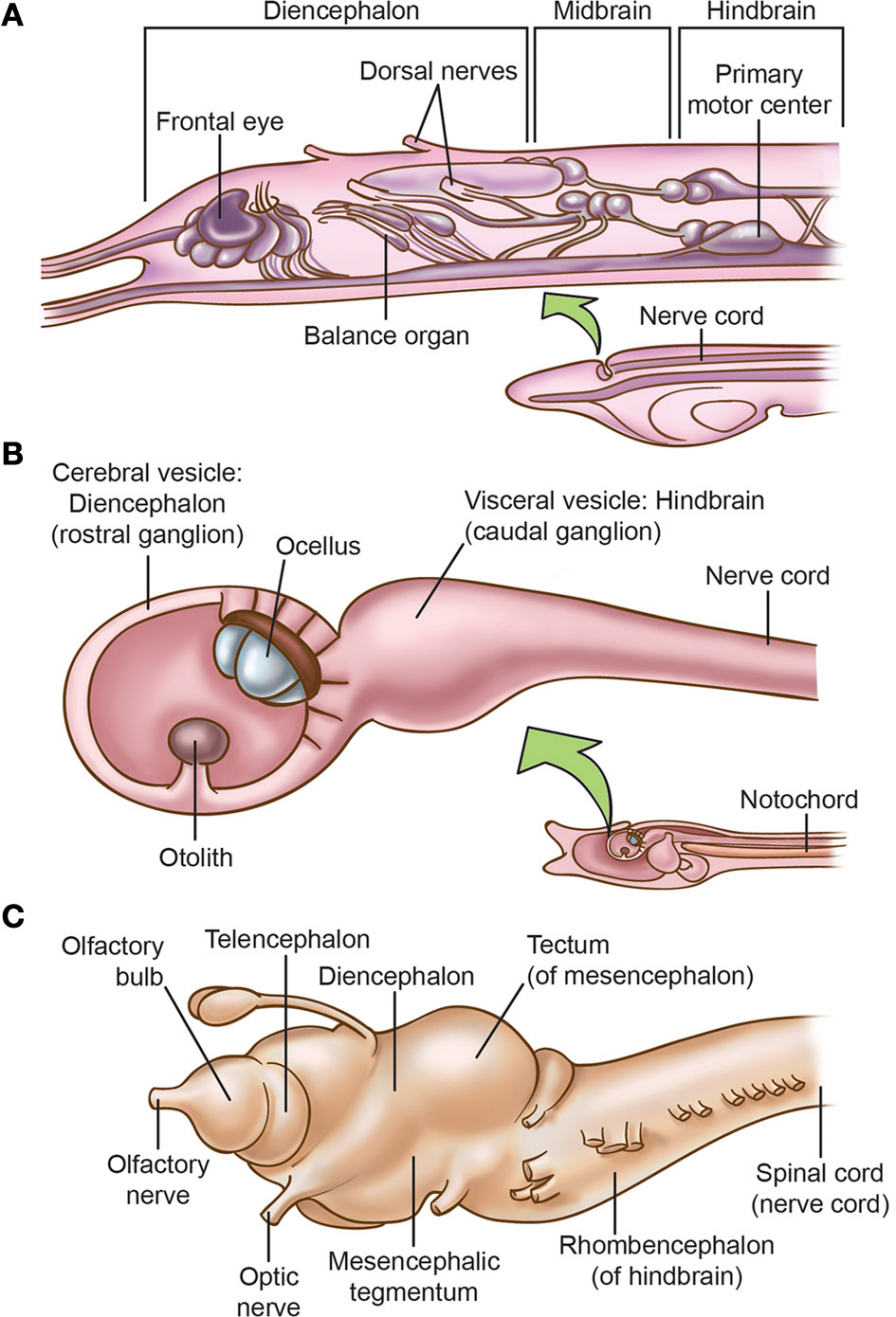
**Comparison of the brains of (A) larval amphioxus, (B) larval tunicate *Ciona intestinalis*, and (C) the lamprey *Lampetra fluviatilis***. In all three pictures, anterior is to the left. Based on Young (1962), Nieuwenhuys (1972, 1977), Burighel and Cloney (1997), Nieuwenhuys and Nicholson (1998), Fritzsch and Glover (2009), and Glover and Fritzsch (2009). Only the lamprey has a well-delineated tripartite brain and the full suite of neural-crest derivatives.

Molecular and neuroanatomical studies indicate that amphioxus has brain structures (Figure 2A) that are homologous to the diencephalic forebrain and the hindbrain of vertebrates, and perhaps also a small midbrain (Lacalli, 1996a,b, 2004, 2005, 2008a,b, 2010; Butler, 2000; Holland and Chen, 2001; Wicht and Lacalli, 2005). For instance, in larval amphioxus the cerebral vesicle located at the rostral end of the neuraxis contains several structures that Lacalli has identified as homologues of diencephalic structures of most craniates, and an unpaired frontal eye in the midline that is the homologue of vertebrates' paired eyes (also see Vopalensky et al., 2012).

Studies suggest that cephalochordates conserve a wider array of primitive chordate characteristics than do tunicates and therefore that amphioxus is the best available model for the proximate ancestor of the vertebrates (Holland and Chen, 2001; Mallatt, 2009; Mallatt and Holland, 2013). In addition, Putnam et al. (2008) support the view that amphioxus reveals the critical features of the genome of the last common ancestor of all chordates and that a pre-Cambrian cephalochordate-like ancestor gave rise to modern cephalochordates as well as to urochordates and vertebrates.

However, Glover and Fritzsch (2009) focus more on urochordates. They note that the free-swimming larvae of most urochordates have a simple central nervous system (Figure 2B) consisting of a rostral ganglion with an ocellus (unpaired eye), a caudal ganglion, and a caudal nerve cord that are homologous to the craniate diencephalon and eye, hindbrain, and spinal cord, respectively. Although the urochordate brain has become specialized and even reduced, Glover and Fritzsch (2009) say it evolved from a more advanced ancestral brain because its bulged sub-parts are more distinct than in the un-bulged, uniformly tube-shaped brain of cephalochordates (compare Figures 2A,B). Further, while it was long supposed that cephalochordates are the closest relatives (sister group) of the vertebrates, more recent molecular-phylogenetic analyses suggest that urochordates instead are the sister group of vertebrates and that the cephalochordates arose earlier (Bourlat et al., 2006; Delsuc et al., 2006; Holland, 2007; Hall, 2008; Lacalli, 2008b; Lamb, 2011; Figure 1).

Protochordates lack some key vertebrate features. Their tiny eyes do not form images (Lacalli, 2004; Lamb et al., 2007, 2008; Lamb, 2011, 2013) and they lack a telencephalic forebrain; thus, the camera eye and telencephalon seem to have been vertebrate innovations (Holland and Chen, 2001; Fritzsch and Glover, 2009; Figure 2C). For now, most experts stick with this straightforward interpretation of the facts (e.g., Lamb, 2013), even though some new genetic and cellular evidence suggests the eyes of both protochordate groups were secondarily simplified, implying the first chordates had slightly more elaborate eyes (Lacalli, 2013; Sestak et al., 2013). Another vertebrate feature is absent from cephalochordates. Although somatosensory cells occur in small clusters on their body surface (Lacalli, 2004), there are no dorsal root ganglia anywhere along the neuraxis (Glover and Fritzsch, 2009). The evidence for an olfactory system is scanty in both groups of protochordates (Lacalli, 2004; Graham and Shimeld, 2012).

In summary, the ancestral chordate nervous system probably resembled that of modern cephalochordates and larval urochordates and featured a primitive brain but lacked a telencephalon. Its eye homologue sensed light but did not form an image. The evolutionary elaboration of these features toward the vertebrate state was the next critical stage in the origins of sensory images and consciousness.

## Date of origin of consciousness

When did this progression toward vertebrates and the hypothesized dawn of vertebrate consciousness occur? The earliest confirmed vertebrate fossils date to 520 million years ago (mya), in the early part of the Cambrian Period (which itself lasted from about 541–488 mya) (Valentine, 2002; Shu et al., 2003; Erwin and Valentine, 2013). Therefore, 520 mya is the most-recent possible date. To deduce the older end of the interval, we note that the first body fossils of any kind of Bilateria are 556 million years old (Erwin and Valentine, 2013), Bilateria being the group of animals that includes the chordates and all the invertebrates except sponges, jellyfish, and their relatives (see Figure 1). This makes 560 mya a reasonable estimate of the maximum age for the vertebrate line, although “molecular clocks” that date the origins of taxa by measuring rates of gene evolution place this earlier, at 605 mya (Erwin et al., 2011). The clock method, however, has been challenged for producing unrealistically early times of origin for animal phyla (Bromham, 2006), even with the refinements used by Peterson et al. (2008), Erwin et al. (2011), and others. Thus, we date the emergence of pre-vertebrates, vertebrates, and their distinctive features to the interval of 560–520 mya (Figure 1).

## Key innovations: neural crest, placodes, and the elaboration of sensory organs

**Figure 3:**
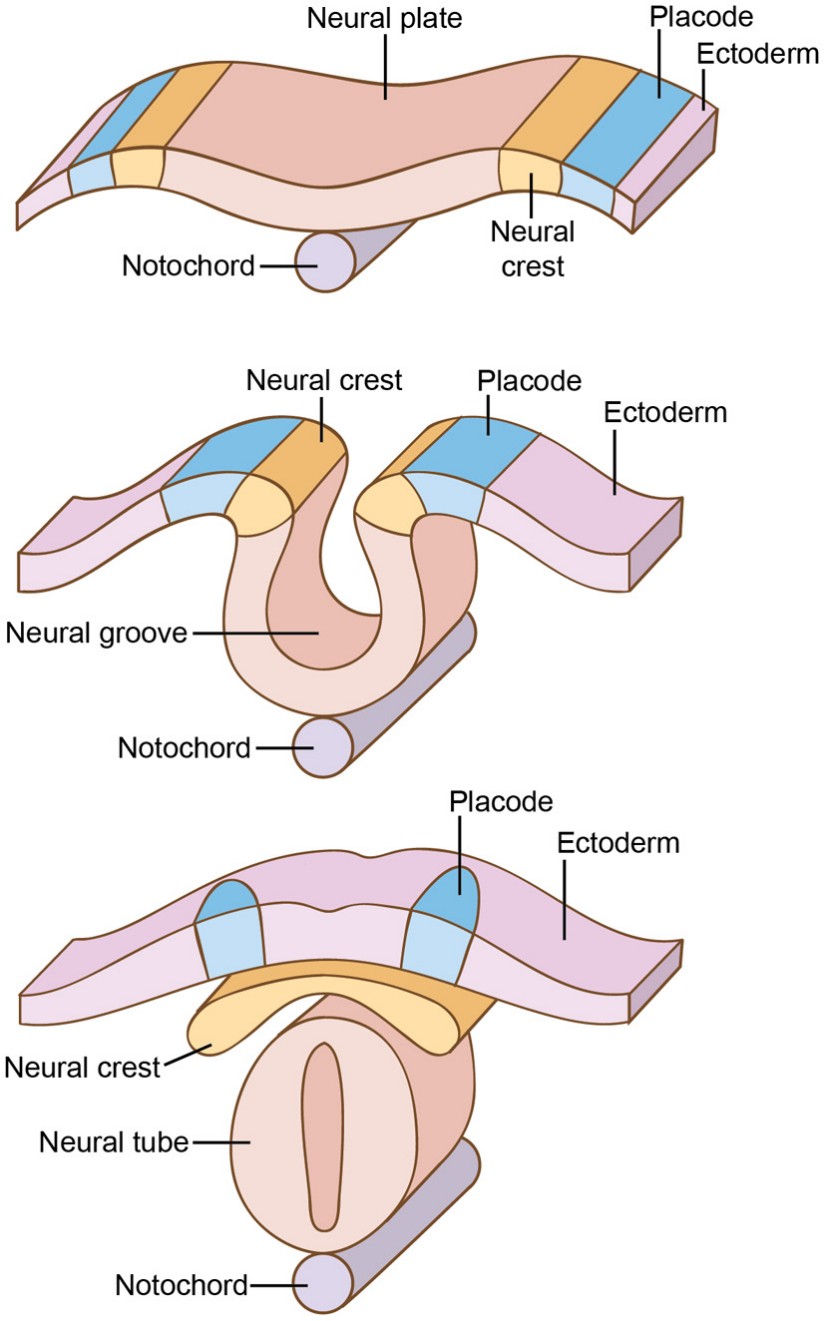
**Development of the neural crest and placodes, near the midline of the back**. In vertebrates, double-walled folds form at the anterior and lateral regions of the neural plate, the inner walls of which give rise to the *neural crest* while the lateral folds give rise to the neurogenic *placodes*.

Perhaps the single most important innovation marking this transition to vertebrates was the appearance of neural crest and neural placodes (Gans and Northcutt, 1983; Northcutt, 1996a,b, 2005; Hall, 2008; Sauka-Spengler and Bronner-Fraser, 2008). The neural plate is a region of thickened ectoderm that forms longitudinally on the dorsal surface of the developing vertebrate embryo (Figure 3). Then, double-walled folds form at the anterior and lateral regions of the neural plate, the inner walls of which give rise to the *neural crest cells* while the lateral folds give rise to the *ectodermal* or *neurogenic placodes* (Allman, 1999; Holland and Chen, 2001; Holland and Holland, 2001; Baker, 2005; Schlosser, 2005, 2008; Donoghue et al., 2008; Hall, 2008, 2009; Graham and Shimeld, 2012).

Neural crest cells migrate into the head and trunk regions of the body where they differentiate into various cell types including some cranial sensory neurons, all the sensory neurons in the trunk, all ganglionic autonomic neurons, and all the pigment cells called melanocytes. Placodes are thickened areas of epithelium that differentiate into neural and non-neural structures. Cranial neurogenic placodes give rise to paired peripheral organs of special sense and contribute to the development of the olfactory system (olfactory placodes that form the olfactory receptors), the lens of the eye, the inner ear (otic placodes that form the hair-cell receptors for hearing and equilibrium), the majority of cranial sensory neurons, and the lateral line system of fish. The neurons in the trigeminal ganglion (cranial nerve V) that transmit sensations of touch, pain, and temperature from the face are of combined neural crest and placodal origin, and the neurons of the facial, glossopharyngeal, and vagus nerves that provide afferent innervation for the taste buds are of placodal origin. Indeed, the entire peripheral nervous system is derived from cells that originate within the neural crest and cranial placodes (Holland and Holland, 2001; Baker, 2005; Schlosser, 2005, 2008, 2010; Donoghue et al., 2008; Hall, 2008, 2009; Graham and Shimeld, 2012). A minor exception is that all life stages of lampreys, and the larvae of fish and amphibians, retain a few non-crest-derived sensory neurons called Rohon-Beard cells. The cell bodies of these exceptional neurons are not in the dorsal roots, but are inside the spinal cord of the central nervous system, mirroring the condition in amphioxus (Nieuwenhuys and Nicholson, 1998; Wicht and Lacalli, 2005; Rossi et al., 2009).

Whether there are neural crest or placodal derivatives in non-craniates is a subject of debate (Glover and Fritzsch, 2009). In protochordates, particularly urochordates, candidate placode- and crest-like cells have been identified, and there is some expression of placode- and crest-specific genes in both cephalochordates and urochordates (Holland and Holland, 2001; Donoghue et al., 2008; Hall, 2008; Holland et al., 2008; Schlosser, 2008; Glover and Fritzsch, 2009; Yu, 2010; Graham and Shimeld, 2012). Recently, Abitua et al. (2012) found the best candidate for a neural-crest homologue in the pigment-forming “A9.49” cells of tunicates, but these cells do not migrate nor form any ectomesenchyme as true crest cells do. According to Glover and Fritzsch (2009), clear-cut, definitive migratory neural crest appears to be absent from cephalochordates and urochordates.

Whatever rudimentary precursors of placodes and neural crest are in fact present in protochordates, the full development of these structures marked a major transition in the evolution of the nervous system. Indeed it has been proposed and largely accepted that the neural crest and placodal systems represent the defining characteristics of the craniate line (Gans and Northcutt, 1983; Northcutt and Gans, 1983; Northcutt, 1996a,b, 2005; Hall, 2008). Kaas (2009) pointed out that the transformation of the neural tube into a fully formed brain coincided with the establishment of all the head's special-sensory systems that are dependent on the neural crest and placodes: image-forming eyes (via the lens), the equilibrium-sensing ears, olfaction, taste and the lateral line of fish.

But these correlated events need not have been *exactly* simultaneous. A recent study of gene expression (Sestak et al., 2013) suggests that the placodes evolved slightly earlier than the neural crest, or at least got a head start (also see Wada et al., 1998). The evidence is that a good number of placode-associated genes are expressed in developing tunicates (even though the adult placodal derivatives are neither obvious nor vertebrate-like in tunicates), but neural-crest-gene expression is high only in the vertebrates.

In summary, along with the evolutionary appearance of placodes and neural crest came the enlarged brain of craniates, well beyond that seen in the protochordates, with a complete craniate forebrain, midbrain, and hindbrain. These brain structures received and processed the input from the crest- and placode-derived peripheral neurons for the senses of olfaction, taste, equilibrium (and later, audition), and for the general somatic senses of the head (Butler and Hodos, 2005). They provided the central territory in which evolved isomorphic sensory maps and images.

## The neurobiology and phylogeny of neural maps and sensory images

### Lampreys as the model for early sensory systems

The lampreys and hagfish (jawless cyclostomes) are considered the most basally arising extant members of the vertebrate clade. Hagfish are secondarily specialized for a deep-ocean burrowing lifestyle (Mallatt, 1997) so the free-swimming lamprey is generally accepted as most closely resembling the first vertebrates in its sensory and brain structures. This highly visual animal (Collin, 2009) reveals that the camera-style eye was present by the time lampreys and gnathostomes diverged over 460 million years ago (Mallatt, 1996; Lamb, 2011; Figure 1). The success of the vertebrate eye stemmed from its innovations that contribute to the higher resolution of a visual image, including a complex, spatially organized three-layered retina; neural transmission from ciliary photoreceptors to chains of output neurons; enlargement of the eye allowed by lateral ballooning of the optic vesicle from the wall of the embryonic diencephalon; the invagination of this ballooned vesicle to form an eye cup; the addition of retinal computing power in the form of retinal bipolar neurons, amacrine neurons, and other interneurons; and the advent of the focusing lens, a placodal derivative (Lamb et al., 2007, 2008; Lamb, 2011, 2013). Along with its elaborate eye and lens, the lamprey has all the other vertebrate features we have been emphasizing: the complete suite of crest and placode derivatives, a well-developed peripheral nervous system and trigeminal ganglia (Murakami and Watanabe, 2009), all the major elements of the tripartite brain (Figure 2C) including a relatively large optic tectum in the mesencephalon, a diencephalon with thalamus and hypothalamus, and a telencephalon that contains an olfactory bulb and a small cerebral hemisphere with a pallium (Nieuwenhuys, 1977; Iwahori et al., 1987, 1999; de Arriba and Pombal, 2007; Fritzsch and Glover, 2009).

### Original roles of telencephalon vs. tectum

Despite the existence of a telencephalon in lampreys and in the reconstructed proto-vertebrate, there has been confusion over how much of the highest-level sensory processing is/was performed in the telencephalon vs. in the optic tectum of the mesencephalon. The classical view was that at first the telencephalon was only a “smell brain” while the tectum was the “visual brain” (Wullimann and Vernier, 2009b). But by the 1970s this view was refuted, largely because technical advances showed the telencephalon of fishes and amphibians to be less olfaction-dominated than was previously thought, and to contain all the same non-olfactory structures as in “higher” vertebrates (e.g., the corpus striatum for selecting and maintaining behavioral actions, the amygdala and other limbic structures for emotions, and the hippocampus for forming memories) (Ebbesson, 1981; Nieuwenhuys and Nicholson, 1998; Grillner et al., 2005; Jacobs, 2012; Kandel et al., 2012; Strausfeld and Hirth, 2013). However, it is still likely that ancestrally, the telencephalon was where smell input was processed and then integrated with information from the other senses, especially in spatial memory-maps in the hippocampal complex that allowed the animal to navigate through space (Jacobs, 2012).

In birds and mammals, the telencephalic *dorsal pallium* performs the highest-order processing of all senses based on isomorphic representations (Wild and Farabaugh, 1996; Wild et al., 1997; Jarvis, 2009; Martinez-Garcia and Lanuza, 2009; Karten, 2013). This pallial zone is especially dominant in mammals as the cerebral cortex (Kaas, 1983, 1997; Thivierge and Marcus, 2007). But in the anamniotes including lampreys, the dorsal pallium lacks this role (except in olfaction) and the optic tectum is the center of sensory isomorphic representations. *This claim is neither original nor disputed* but is widely accepted among fish and amphibian researchers (Schuelert and Dicke, 2005; Binder et al., 2009; Mueller, 2012). The tecta of non-mammalian vertebrates have multiple isomorphic maps. The *retinotopic* maps closely overlap auditory *tonotopic* maps, *vestibular* maps, *lateral-line*-receptive maps, and *somatosensory* somatotopic maps (Sparks and Nelson, 1987; Sparks, 1988; Stein and Meredith, 1993; Hodos and Butler, 1997; Merker, 2007, 2012; Braun, 2009; Saidel, 2009; Cornide-Petronio et al., 2011; Stephenson-Jones, 2012). Given this, we deduce that the optic tectum, not the pallium, is the main site of sensory images and hence consciousness in anamniotes. Merker (2005) deduced this from much the same evidence, and he also emphasized the tectum's laminar organization, which allows efficient and extensive integration of the isomorphic input from the different senses, and emphasized that the tectum receives multisystem convergence from many other parts of the brain (de Arriba and Pombal, 2007).

Additional support for the role of the anamniote tectum comes from Dicke and Roth (2009) who stated, “In amphibians, as in all anamniote vertebrates…, the tectum is the major brain center for integrating visual *perception* and visuomotor functions. In the amphibian tectum, localization and *recognition* of objects and depth perception takes place. Three separate retino-tectal subsystems for object recognition exist, which process information about (i) size and shape, (ii) velocity and movement pattern and (iii) changes in ambient illumination. These kinds of information are processed at the level of different retinal ganglion cells and tectal neurons in close interaction with neurons in other visual centers.” In this passage, we italicized the words that suggest visual consciousness, which was also implied by Wullimann and Vernier (2009a) when they said the fish tectum is for “object identification and location,” and by Dudkin and Gruberg (2009) when they said the tectum is for “discriminating between different classes of objects, selecting (or attending to) one of several objects, and directing movement of eye or head or body.” In describing the role of the anamniote tectum in multimodal sensory processing, Saidel (2009) said, “Among poikilothermic vertebrates, the tectum has a coordinated map of space resulting from at least two if not more senses that contribute to the transformation of synaptic connections into a sensory map. The tectum might be considered as a two-dimensional [grid] whose coordinate points, determined from the visual field, both specify the external influences and personal space so that the appropriate action is spatially determined. This would be the underlying basis of orientation behavior.”

Regarding the lack of any mammal-like isomorphic maps in the telencephalic pallium of lower vertebrates, Wilczynski (2009) wrote, “… all sensory systems not just olfaction reach large areas of the telencephalon. In this general way, amphibians are similar to other tetrapods, notwithstanding that the inputs are dominated by a very heavy middle thalamic input to the striatum. *The details however, reveal a quite different functional organization from that which might be expected*… The telencephalic targets of ascending sensory pathways are all multimodal. There is no evidence for separate representations for each sensory system, no indication of a topographically preserved projection from any thalamic nucleus to any telencephalic area and *no physiological evidence for a sensory (or for that matter motor) map*. *In essence, there is no evidence for the distinct, unimodal, mapped sensory representations that are so prominent in the mammalian cortex*. A possible exception may be the core olfactory-recipient regions of the lateral pallium. That is not to say that there is or is not a homologue of mammalian neocortex within the amphibian telecephalon, but there are certainly no functional equivalents for the well-mapped, pure sensory zones that are so prominent in mammals and are significant telencephalic components in reptiles and birds” (*emphasis added*). Though Wilczynski spoke of amphibians, this is also true for the pallium of most fish (Wullimann and Vernier, 2009b; but see Prechtl et al., 1998). But the lack of pallial isomorphism is even more extreme in a few, exceptional, fish. For instance, in zebrafish and goldfish of the carp family, neither the visual nor the auditory sensory pathway seems to reach the dorsal pallium *at all* (Mueller, 2012). In conclusion, it is widely accepted that in anamniotes the neurological basis for all sensory representations except olfaction is within the optic tectum not the dorsal pallium.

### Importance of vision compared to the other senses

The importance of the visual tectum in lower vertebrates suggests that visual representations were important in the earliest stages of vertebrate evolution. All the sensory systems are remarkably conserved across the vertebrates, and each resembles the visual system in its basic organization (Shepherd, 1974; Pallas, 1990; Hodos and Butler, 1997). Thus, the monopolar or pseudomonopolar neurons of the olfactory, somatosensory, gustatory and auditory receptors match the bipolar neurons that innervate the rods and cones in the visual system in that all terminate on primary central sensory neurons called first order multi-polar neurons (Butler, 2000); and in most cases these first-order neurons project to the optic tectum and the thalamus (Table 1). Further, Shepherd notes that all the principles involved in the formation of an *initial visual image* in the retina, including “the initial image representation in a two-dimensional sheet, lateral inhibition, temporal transients, contrast enhancement, center-surround inhibition, and feature extraction” (Shepherd, 2012, p. 65) also play essential roles *in the formation of neural “ images” in every other sensory system*. For example, in audition, individual nerve fibers from the ear carry information that has an optimal sound frequency, and lateral inhibition between fiber pathways sharpens the response to that frequency. The same is true in the sense of touch, where tactile discrimination depends upon the density of innervation of the skin and lateral inhibition within central pathways (Shepherd, 2012).

Olfaction, however, shows some fundamental differences. Not only is olfaction not processed by the midbrain tectum, but also it is the only sense in vertebrates that reaches the pallium and subpallium without an obligatory relay through the thalamus (Gottfried, 2006, 2007; Shepherd, 2007, 2012; Table 1). Why the olfactory system does not require a thalamic relay is an interesting question. Gottfried (2006) suggested it is because the olfactory pathways evolved before the emergence of the thalamotelencephalic pathways for the other senses. Whatever the explanation, this fact underscores that a thalamic relay is not a mandatory requirement for the presence of a conscious sensory image (Shepherd, 2007, 2012).

The olfactory system of vertebrates forms an early smell representation—which is comparable to a retinal visual representation—at the level of the glomeruli within the olfactory bulb (Leon and Johnson, 2003; Gottfried, 2010; Shepherd, 2012). From there, serial processing through the primary olfactory cortex and then the orbitofrontal cortex forms a conscious smell image (Tanabe et al., 1975; Zatorre and Jones-Gotman, 1991; Gottfried, 2006, 2007; Shepherd, 2007; Li et al., 2010; Table 1). The primary olfactory cortex is the highest *unimodal* region in the olfactory pathway. Multimodal are the aforementioned hippocampal maps (Jacobs, 2012) and the orbitofrontal cortex. The latter is the main neocortical recipient of projections from the olfactory cortex and has been posited to play a pivotal role in olfactory associative consciousness (Li et al., 2010; Shepherd, 2012). It serves as an integration zone for olfactory afferents with other sensory afferents (Price, 2007).

Although this description is based on mammalian studies, lampreys and other anamniotes have homologous structures throughout their olfactory pathway: the olfactory bulb, then to the “primary olfactory cortex” as their lateral pallium, which then projects to the orbitofrontal-cortex homologue, namely to a part of the dorsal pallium (Northcutt and Wicht, 1997; Wullimann and Vernier, 2009b). From these similarities, we deduce that the telencephalic pallium was a center of sensory (olfactory) consciousness in early vertebrates, even though the optic tectum dominated for the other conscious senses.

Nociception is an important sense to consider, given the current scientific and popular interest in whether lower vertebrates such as fish feel pain (Sneddon et al., 2003; Sneddon, 2004, 2011, 2012; Braithwaite, 2010). *Nociception* and *pain* are related phenomena but are not identical. Nociception is a neurobiological term that involves the neural processing of particular forms of noxious stimuli that could cause tissue damage to the animal. The International Association for the Study of Pain defines pain as “an unpleasant sensory and emotional experience associated with actual or potential tissue damage, or described in terms of such damage” (Nordgreen et al., 2009; Sneddon, 2011). Nociception may be reflexive, and only necessarily involves nociceptive neural pathways; pain is a psychological state, and entails sensory, phenomenal consciousness (see *Introduction*). The most parsimonious assumption is that the principles involved in the production of “pain images” are the same as those involved in the production of other sensory images such as visual images, auditory images, etc.

**Figure 4:**
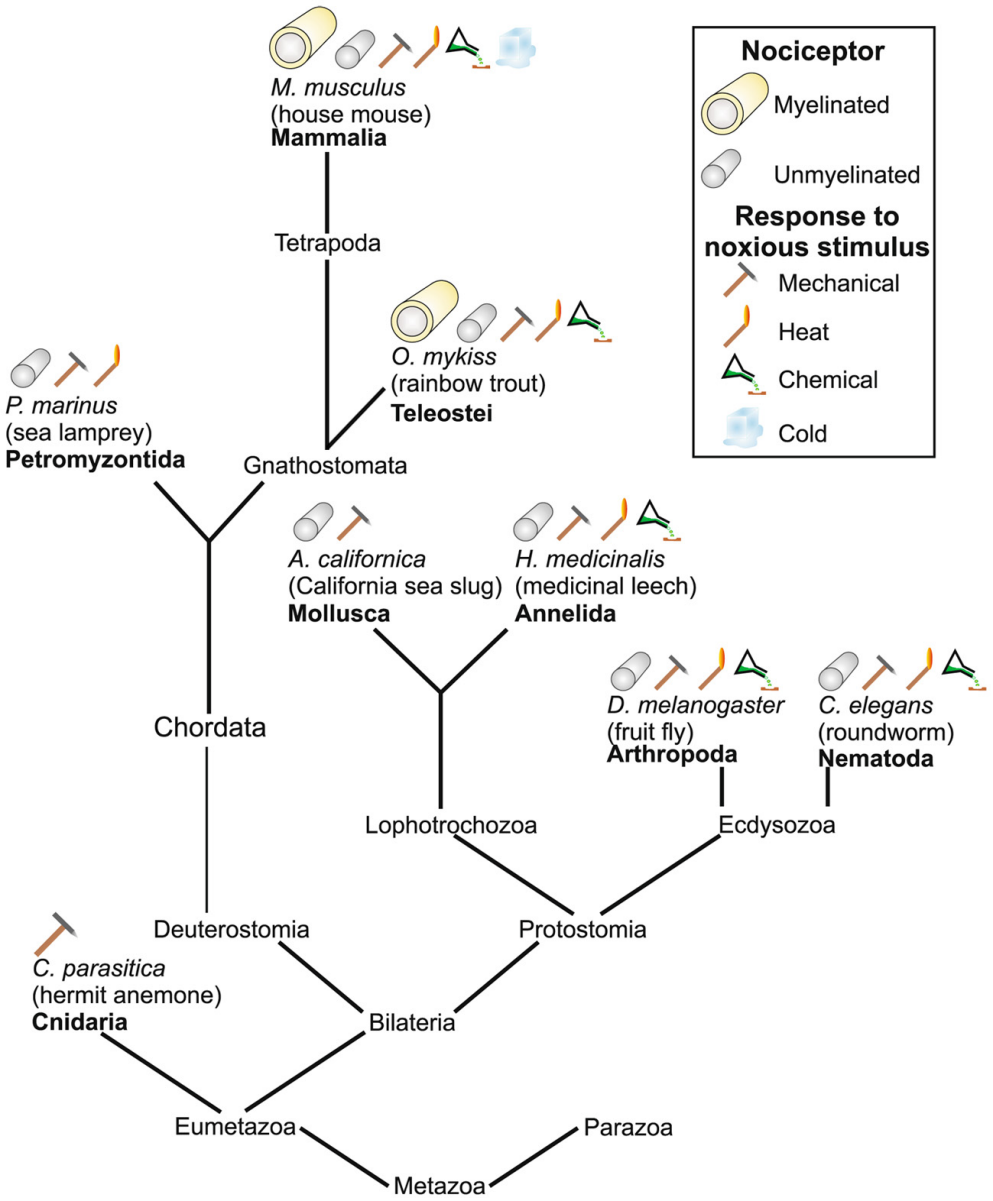
**Various types of nociceptors found across different species**. From Figure 4 in Smith and Lewin (2009) Springer. Reprinted with kind permission from Springer Science+Business Media B.V.

While pain is a complex and multidimensional sensory experience based upon hierarchical somatosensory, affective, and homeostatic information processed in parallel and overlapping brain networks (Craig, 2002, 2003a,b,c; Brooks et al., 2005), nociceptors are actually quite ancient neural structures and are present in species of molluscs, nematode worms, and fruit flies (Smith and Lewin, 2009; Figure 4). In vertebrates, nociceptive neurons with cell bodies in the dorsal root ganglia and innervating the postcranial part of the body are of neural-crest origin, while those innervating the face are in the trigeminal ganglia and derive from both neural crest and placodes (Fitzgerald, 2005; George et al., 2007, 2010; Shiau et al., 2008).

While little is known about lampreys' nociceptive abilities, what is known suggests their nociception is primitive compared to their other senses. The peripheral nervous system of the lamprey has no myelinated nociceptive fibers, and recordings from spinal and brain dorsal cells that have a potential nociceptive role have achieved mixed results (Martin and Wickelgren, 1971; Rovainen and Yan, 1985; Smith and Lewin, 2009). However, Matthews and Wickelgren (1978) reported finding nociceptive neurons in the lamprey trigeminal ganglia. Overall, these preliminary findings suggest nociception is not robust in comparison to its other sensory systems.

However, in the advanced bony fish (teleosts) such as trout, zebrafish, carp, and perch, nociceptor fibers are comparatively well-developed (Sneddon, 2003, 2004, 2011, 2012; Sneddon et al., 2003; Nordgreen et al., 2009; Smith and Lewin, 2009; Braithwaite, 2010) and there is evidence for many of the structures thought to be crucial for the central processing of pain (Figure 4). For instance, in trout and goldfish, Dunlop and Laming (2005) found that responses to both mechanoceptive (brush) and nociceptive (pin-prod) stimuli ramified widely along the neuraxis including the spinal cord, cerebellum, tectum, and telencephalon. Trigeminal afferents in the hindbrain of goldfish show a clear pattern of descending pathways and a topographical organization similar to that present in higher vertebrates (Puzdrowski, 1988). Furthermore, in a host of teleosts, including trout, goldfish, and zebrafish, there is solid behavioral evidence for sustained (in some cases for hours), complex, and goal-directed responses to pain. These responses include rubbing the skin at the site of an injection of a noxious substance, reduction in typically ongoing behavior such as feeding, avoidance of areas where painful stimuli were administered, and inattention to competing stimuli (Sneddon, 2003, 2004, 2011, 2012; Dunlop and Laming, 2005; Ashley et al., 2007; Millsopp and Laming, 2008; Reilly et al., 2008; Braithwaite, 2010; Roques et al., 2010). Reilly et al. (2008) reported two of five common carp rubbed their lips against the tank walls after topical injection of acetic acid into the lips.

Thus, the pain experience seems to characterize teleost fish and presumably all other vertebrates with equally or more complex brains (except perhaps for the cartilaginous fish such as sharks, which may lack appreciable perception: Smith and Lewin, 2009; Sneddon, 2011). That is, the evidence suggests pain occurs in the bony vertebrates that share the necessary neural crest- and placode-derived nociceptors, the brain processing-centers, and behaviors associated with pain processing. Teleosts may not have the entire “pain hierarchy,” however, because the requisite sensory-somatotopy probably does not extend up to the pallium (see above; Wilczynski, 2009). In contrast, in humans and other primates, pain perception has a large cortical component, and there are two candidate cortical regions for somatotopically mapped nociception—the primary somatosensory cortex (SI) that emphasizes the exteroceptive aspects of pain perception (Kenshalo et al., 2000; Mancini et al., 2012), and the insula, which emphasizes its interoceptive aspects (Sherrington, 1906; Craig, 2002, 2003a,b,c, 2009, 2010; Price et al., 2003; Feinberg, 2009, 2011).

## The evolution of image-forming eyes, the cambrian explosion, and the first conscious vertebrates

In the timeline proposed by Lamb and co-workers (Lamb et al., 2007, 2008; Lamb, 2011, 2013), beginning about 600 mya the eye started to evolve from the simple frontal eye characteristic of cephalochordates into the vertebrate camera-style eye that was fully developed by 500 mya and was similar to that of the modern day lamprey. This is also our view, although as mentioned, we put the interval at 560–520 mya (Figure 1). The frontal eye of larval amphioxus, limited to a few dozen cells, provides information about the distribution of light and dark in the surroundings and serves as a light and shadow detector, visual functions most likely involved in establishing the animal's orientation in the water during feeding (Lacalli, 1996b, 2004). However, this lens-less eye does not have the anatomy required for image-formation or complex pattern-recognition; for example, for the detection of prey or to guide complex locomotion (Lamb et al., 2007, 2008; Fernald, 2009; Lamb, 2011).

Attaining image-forming eyes had profound implications for the evolution of animal groups. According to the “Light Switch” hypothesis proposed by Parker (2003), the nearly simultaneous appearance of image-forming eyes in numerous phyla led directly to the diversification of the bilaterian animals during the Cambrian explosion approximately 541 million years ago. In this account, pre-Cambrian animals possessed primitive chemoreceptors and simple light receptors as exist in amphioxus, but it was the evolution of image-forming eyes that led to the explosive improvements in directed locomotion and food seeking, food handling, predation and avoidance of predators, and the origin of hard body parts as defense against predators. Trestman (2013) recently fleshed out the Light Switch hypothesis by detailing how the appearance of object-oriented, spatial vision led not only to brain elaboration but also to a basic kind of “cognition” that controlled the body actions of locomotion and feeding. Further buttressing the Light Switch and vision-first hypothesis, many new retina-associated genes seem to have been added in the earliest Bilateria and their immediate descendants (Sestak et al., 2013).

The centrality of vision in the evolution of the vertebrate brain finds support from studies of embryonic development and cellular differentiation. For instance, although the fates of placodal structures are varied, and they contribute to multiple special sensory structures including the eye lens, inner ear and olfactory epithelium, Bailey and co-workers (2006) found that in the chick embryo, the entire preplacodal region is initially specified as lens tissue, a finding that implies that “lens” is the default state of the preplacodal territory and that all the non-lens placodal derivatives, such as those contributing to the inner ear, evolved later.

**Figure 5:**
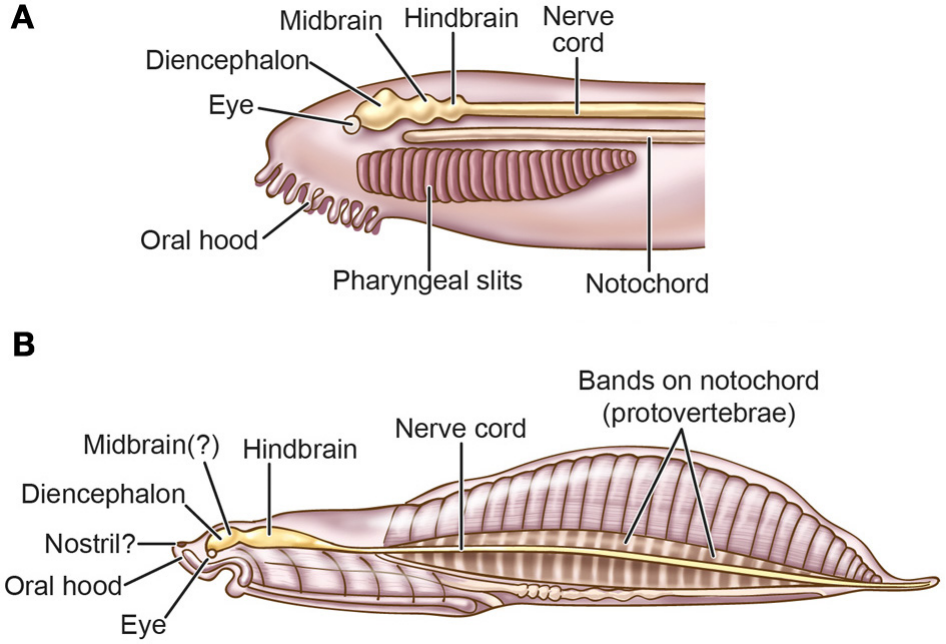
**Nervous systems of pre-vertebrates. (A)** The “cephalate” as hypothesized by Butler (2000, 2006; Butler and Hodos, 2005). **(B)** *Haikouella*. This fossil animal is interpreted to have had paired eyes, less prominent or absent olfactory organs, a poorly developed telencephalon, and no otic or vestibular organs (Mallatt and Chen, 2003; Chen, 2009, 2012). Mallatt and Chen (2003) propose that *Haikouella* supports Butler's model of the hypothetical cephalate.

In her intriguing scenario of the evolution of vision and the brain, Butler (Butler, 2000, 2006; Butler and Hodos, 2005) hypothesized how the advent of an advanced visual system played an early and critical role in the formation of the vertebrate brain. She proposed that there was a transitional animal between a cephalochordate-like ancestor and the first true craniates, which she called a “cephalate” (a combination of the words “cephalochordate” and “craniates”). This hypothetical creature (Figure 5A) had paired eyes and a fairly well-established diencephalon- and mesencephalon-based visual system (note that the vertebrate retina is embryologically a direct outgrowth of the diencephalon), but at this early stage it lacked most of the contributions from neural crest and placodes, and lacked a craniate-type peripheral nervous system and a definitive telencephalon. According to this account, the transition from cephalochordate to craniate was sequential, beginning with the establishment of paired, lateral eyes and optic nerves followed by elaboration of the descending visual pathway to brainstem motor centers. These visual pathways served as a *circuitry template* for the subsequent arrival of the new sensory systems, both ascending and descending, that evolved with the advent of the neural crest and placodes including the somatosensory, olfactory, otic-equilibrium, and gustatory systems. She argued that this model explains the marked uniformity of the pattern across the different central-sensory pathways of vertebrates (Table 1). Butler's hypothesis suggests to us that the appearance of the *visual image* was the earliest manifestation of sensory consciousness, followed by others.

This eye-first view could be questioned. For example, Plotnick et al. (2010) reasoned that *olfaction* evolved first, on mostly theoretical-ecological grounds. But the actual evidence favors eye-first. Sestak et al. (2013) found that the surge of newly evolved retina- and lens-associated genes pre-dated that of new olfactory, otic, and lateral-line genes, having occurred before, vs. after, the appearance of the “tunicate + vertebrate” line of animals. Additionally, Vopalensky et al. (2012) used gene-expression patterns to show that larval amphioxus has photoreceptors, pigment cells, and projection neurons that are homologous to those in the retina of vertebrates, yet amphioxus lacks vertebrate-like olfactory and equilibrium sensors. Third, fossil pre-vertebrates show more evidence of eyes than of olfactory organs, as we will now discuss.

Evidently supporting the cephalate model are the fishlike, fossil *yunnanozoans*, from the Yunnan Province of China, of which *Haikouella lanceolatum* (Figure 5B) is known in the most detail (Chen et al., 1999; Chen and Li, 2000; Mallatt and Chen, 2003; Mallatt et al., 2003). *Haikouella* dates from the Early Cambrian 520 mya, not long after the hypothesized emergence of visual pre-vertebrates at 560–520 mya. *Haikouella* was 25–30 mm long and possessed a notochord, paired eyes, a prominent hindbrain, and a diencephalon located in the same positions as are these structures in extant vertebrates such as the lamprey. As interpreted by Mallatt and Chen (2003), *Haikouella* had no skull or ears, and it had at most a weakly developed telencephalon. This brain region may require a fully developed olfactory placode for its induction (Butler, 2000, 2006; Butler and Hodos, 2005), which would imply that *Haikouella* had only a tiny or non-existent olfactory placode; indeed, only hints of olfactory capsules and nostrils are seen in the adult fossils. Thus, *Haikouella*, despite having the vertebrate eyes, appears to have lacked many of the skeletal and peripheral-nervous components that are present in vertebrates with fully evolved neurogenic placodes and neural crest.

But *Haikouella* had gill bars, which are neural-crest derivatives, and in the center of each eye, a dot-like lens, which is a placode derivative (Figure 7 in Mallatt and Chen, 2003). With its eyes, placodes and neural crest, and a brain whose overall size matches that of modern vertebrates (i.e., lampreys), *Haikouella* is a candidate for the earliest conscious organism on earth, or at least the earliest conscious chordate.

**Figure 6:**
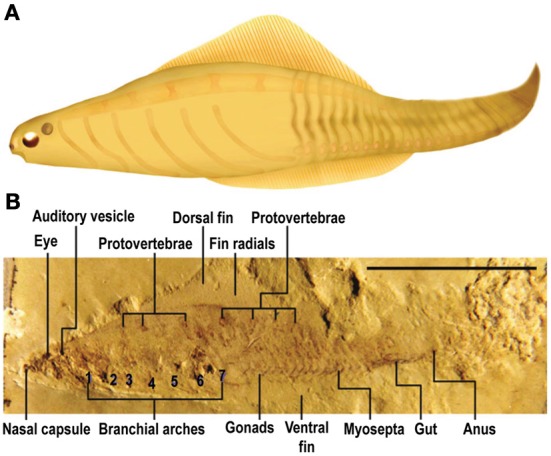
***Haikouichthys*. (A)** Artist's rendering of what *Haikouichthys* looked like. **(B)** Fossil of this animal with an eye and otic capsule (“Auditory vesicle”) labeled. *Haikouichthys* is agreed to have been a true vertebrate, a jawless fish, and it shows vertebral elements (protovertebrae), prominent eyes, and nasal capsules (Shu, 2003; Shu et al., 2003, 2009). From Figure 146 in Chen (2012) Springer. Reprinted with kind permission from Springer Science+Business Media B.V.

It should be noted, however, that the interpretation of the *Haikouella* fossils is not without controversy. Most prominently, Shu and co-workers have questioned *Haikouella*'s evolutionary placement and even the existence of eyes, a notochord, or a brain in this animal (Shu et al., 1996, 2009; Shu, 2003; also see Donoghue and Purnell, 2009). This leaves another 520-million-year-old group from the same fossil beds, *Haikouichthys ercaicunensis* (Figure 6) and related species, as best indicating the early evolution of the vertebrate nervous system (Shu et al., 1999, 2003, 2009; Hou et al., 2002; Shu, 2003; Chen, 2012).

*Haikouichthys* is widely agreed to have been a true vertebrate, a jawless fish, and it shows vertebral elements, prominent eyes, and otic and olfactory capsules, although no trace of *Haikouichthys*' brain has been preserved in the fossils. As a vertebrate, *Haikouichthys* would be a more evolved species than *Haikouella* (Chen, 2012) and is therefore possibly less informative about the features of a putative transitional pre-craniate. Nonetheless, whichever of these fossil groups represents the progenitor of craniates (or is an early craniate), the paired eyes of *Haikouella*, other *yunnanozoans*, and *Haikouichthys* ranged in size from 0.2 to 0.6 mm in diameter, and thus were considerably larger than the frontal eye of amphioxus, which is only 10 microns in diameter. This suggests that the eyes of these fossil animals had more neuronal layers, forming two-dimensional receptor fields that produced retinal images, at least in the larger-eyed *Haikouichthys* (Chen, 2012). Based upon our aforementioned criteria, *Haikouichthys* possessed primary consciousness.

## The simplest extant vertebrates with consciousness: the argument for the lamprey

In our hypothesis, the creation of a sensory neural map requires at a minimum a brain and typical neurohierarchical structure, with consciousness emerging from progressively more complex and integrated patterns of isomorphic organization in the upper levels of this hierarchy (Tables 1, 2; Feinberg, 2009, 2011, 2012). But the brain-maps for the different senses are not isolated from one another. They are integrated in two critical ways. First, the highest levels are multimodal; for example, the visual, auditory, vestibular, and somatosensory maps all stack in register in the midbrain tectum (Guirado and Davila, 2009; Saidel, 2009); as another example of this, extensive multimodal association-areas and association fibers interlink the different primary sensory-areas in the cerebral cortex of mammals (Mesulam, 2000; Feinberg, 2011). The second critical feature of the neural correlates of consciousness is widespread interaction among such separate brain regions as the reticular-activating system (RAS) of the reticular formation, the thalamus, and the optic tectum or cerebral pallium/cortex, as these brain regions receive and integrate sensory representations into neural networks that contribute to attention, awareness, and neural synchronization (Penfield, 1975; Baars, 1988, 2002; Edelman, 1989, 1992; Newman and Baars, 1993; Crick, 1994; Llinas and Ribary, 2001; Ribary, 2005; Seth et al., 2005; Min, 2010; Edelman et al., 2011). Therefore, for a cyclostome brain to create sensory mental images, its isomorphic representations must be integrated into this wider neural network. In fact, there is ample evidence that the most studied cyclostome nervous system of the lamprey satisfies this second requirement (Nieuwenhuys, 1972, 1977; Polenova and Vesselkin, 1993; Northcutt and Wicht, 1997; Nieuwenhuys and Nicholson, 1998), as will now be elucidated.

**Table 2:** **Neural features, functions, and genes proposed to contribute to consciousness in vertebrates**.

**Feature**	**Function**	**Genes involved**	**References**
Paired lateral eyes	Gather visual images, guide vision-related actions. Retinas of these eyes develop from the diencephalon and co-evolve with a tripartite brain	*Pax4/6* (etc.)	Shu et al., 1999, 2003, 2009; Butler, 2000; Collin et al., 2003^*^; Vopalensky et al., 2012; Lamb, 2013
Fully differentiated tripartite brain	Provides for a sensory-neural hierarchy up to pallium or cerebral cortex (amniotes), or to optic tectum and thalamus (anamniotes)	*Emx* (dorsal pallium) *Otx2*, *Fgf8*, *En*, etc. (tectum)	Friedman and O'Leary, 1996; Nieuwenhuys and Nicholson, 1998^*^; Butler, 2000, 2006; Murakami and Kuratani, 2008^*^; Rhinn et al., 2009; Sprecher, 2009
Placodes and neural crest	Provide lens of eye and the lower levels of the neural hierarchies of all major isomorphic sensory systems in vertebrates, except the visual		Holland and Holland, 2001; McCauley and Bronner-Fraser, 2002^*^, 2003^*^; Baker, 2005; Schlosser, 2005, 2008, 2010; Donoghue et al., 2008; Hall, 2008, 2009; Graham and Shimeld, 2012
Placode genetics		*Six1*, *Six4*, *Eya* (all placodes), *Robo2* (trigeminal ganglion), *Pax* genes including *Pax6* (lens)	McCauley and Bronner-Fraser, 2002^*^; Bailey et al., 2006; Schlosser, 2008; Shiau et al., 2008; Yu, 2010
Neural crest genetics		*Snail1/2, FoxD3, Twist, Hoxb2, Hoxa2, Hoxb3, Hoxa3, Slit1*	Trainor and Krumlauf, 2000; Murakami and Kuratani, 2008^*^; Schlosser, 2008; Shiau et al., 2008; Yu, 2010
Reticular activating system (RAS)	Widespread brain activation mediating attention and arousal		Moruzzi and Magoun, 1949; Parvizi and Damasio, 2001; Dehaene et al., 2003; Manger, 2009
Reciprocal tecto-thalamic interactions	Proposed integration of higher order sensory representations		Heier, 1948^*^; Nieuwenhuys and Nicholson, 1998^*^; Merker, 2005, 2007
Isomorphic neural representations	Provide for the spatial or non-spatial mapping of the external or internal environment		Hamdani and Doving, 2007^*^; Murakami and Kuratani, 2008^*^; Cornide-Petronio et al., 2011^*^; Kandel et al., 2012; Stephenson-Jones, 2012^*^
Isomorphic genetics		*Hoxa2, EphrinA, EphrinB, Tnc, Nov, Slo, En-1, En-2*	Friedman and O'Leary, 1996; McLaughlin and O'Leary, 2005; Gosse et al., 2008; Murakami and Kuratani, 2008^*^; Bevins et al., 2011; Frucht et al., 2011; Son et al., 2012
Color vision	Provides for the phenomenal/subjective representation of different light-wavelengths	Opsins	Jacobs, 2009; Lamb, 2013^*^
Non-visual sense organs (olfaction, taste, somatosensory, equilibrium and hearing, lateral line, electroreception)	Various chemosensory, mechanosensory, and electrosensory functions. Well-developed olfactory sense can guide complex food-finding and migratory patterns	OR, TAAR, V1R (olfactory), TR, PKD2L1 (taste), various ion-channel genes (hearing, equilibrium, touch, pain)	Braun and Northcutt, 1998^*^; Vrieze and Sorensen, 2001^*^; Shu, 2003^*^; Shu et al., 2003^*^; Chung-Davidson et al., 2004^*^; Chandrashekar et al., 2006; Libants et al., 2009^*^; Niimura, 2009; Vrieze et al., 2010^*^; de Brito Sanchez and Giurfa, 2011; Horwitz et al., 2011; Kawashima et al., 2011; Geffeney and Goodman, 2012; Roudaut et al., 2012; Baker et al., 2013

Asterisks

(*) *indicate the references that specifically document the features in lampreys and other fish*.

**Figure 7:**
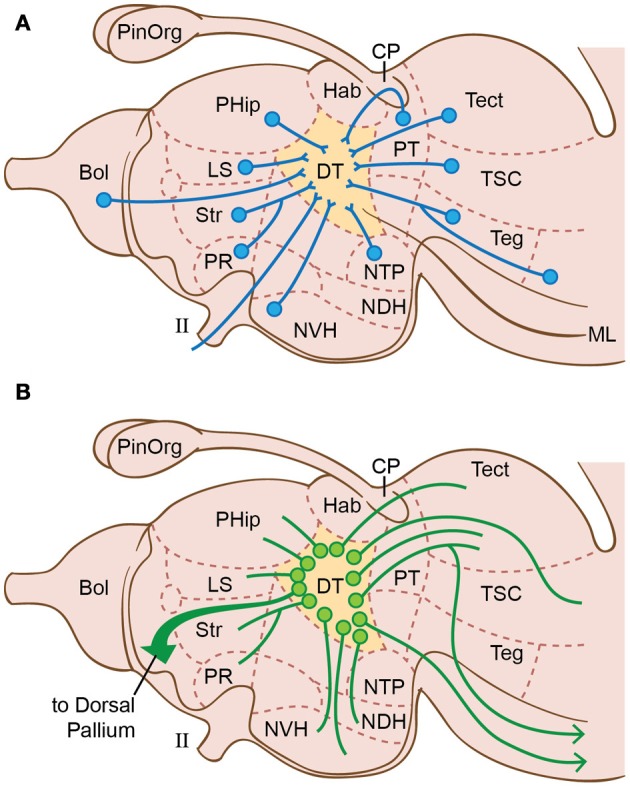
**Brain regions in lampreys, emphasizing the connections of the dorsal thalamus (DT) according to Nieuwenhuys (1972); Nieuwenhuys (1977) and Nieuwenhuys and Nicholson (1998). (A)** Afferents to dorsal thalamus. **(B)** Efferents from dorsal thalamus. II, optic nerve; Bol, olfactory bulb; CP, commissura posterior; Hab, ganglion habenulae; LS, lobus subhippocampalis; ML, medial lemniscus; NDH, nucleus dorsalis hypothalami; NVH, nucleus ventralis hypothalami; NTP, nucleus tuberculi posterioris; PinOrg, pineal organ; PHip, primordium hippocampi (= hippocampus, medial pallium); PR, nucleus preopticus; Str, corpus striatum; PT, area pretectalis; Tect, tectum mesencephali; Teg, tegmentum; TSC, torus semicircularis.

The lamprey nervous system possesses every typical sensory-integration center of vertebrates including the optic tectum, the dorsal thalamus, the RAS in the tegmentum, the telencephalic pallium and olfactory bulb (Figure 7) (Heier, 1948; Wicht, 1996; Nieuwenhuys and Nicholson, 1998). The lamprey brain also has the widespread interactions and neurohierarchical properties that we consider necessary for sensory consciousness. Let us examine the integration centers one by one.

We already mentioned the optum tectum's role in sensory integration and isomorphic-map formation in the lamprey (Saidel, 2009; Stephenson-Jones, 2012), which would make it the key contributor to all non-olfactory sensory consciousness. To expand on this consideration of the tectum in consciousness, the nearby *isthmus nucleus* has now been identified in lampreys (Robertson et al., 2006). The isthmus nucleus signals the tectum to direct *attention* to important objects in the visual field (in birds, see Wylie et al., 2009), or at least it *arouses* and *alerts* the system that something is moving in the field (in teleosts: Dudkin and Gruberg, 2009). Either of these roles could be a part of conscious perception.

Turning to the dorsal thalamus of lampreys (Figure 7), Heier (1948) and Nieuwenhuys and Nicholson (1998) concluded this is another higher-order integration center for correlating various types of afferent information. That is, it integrates information from the tectum (vision and most other senses), olfactory bulb (smell), and the spinal cord and rhombencephalon (the somatosensory information carried by the spinothalamic tracts and trigeminal nuclei). In this way, the thalamus would interact with the tectum to help generate tectal consciousness. This simple idea is similar to Merker's (2005, 2007) more complex interpretation, but without the latter's radical claim that the center of consciousness in *mammals* is the brainstem instead of the cerebral cortex (cf. Crick, 1994; Fries et al., 1997; Zeki and Marini, 1998).

Along with its tectal interactions, the dorsal thalamus of vertebrates is the station for reciprocal communications between the telencephalon and the rest of the brain (Butler, 2008b). As in other vertebrates, the lamprey's dorsal thalamus projects to the cerebral pallium (Figure 7B) indicating a pallial role in sensory processing (Polenova and Vesselkin, 1993; Northcutt and Wicht, 1997).

Additionally, the lamprey brain possesses a well-developed reticular formation with extensive reciprocal connections to the dorsal and ventral thalamus, the latter actually including the most rostral part of the reticular formation (Stefanelli, 1934; Heier, 1948; Nieuwenhuys and Nicholson, 1998; Butler, 2008b). In all vertebrates, the reticular formation signals widespread activation of the cerebrum and thus is required for alertness, awareness, attention, and consciousness (Moruzzi and Magoun, 1949; Parvizi and Damasio, 2001; Dehaene et al., 2003; Manger, 2009). In lampreys and other anamniotes, this RAS could activate the tecto-thalamic sensory consciousness that we have proposed. In mammals, the system is elaborated into a reticular formation-thalamocortical complex that is essential for mammals' cerebrum-dominated consciousness (Baars, 1988, 2002; Edelman, 1989, 1992; Newman and Baars, 1993; Crick, 1994; Llinas and Ribary, 2001; Ribary, 2005; Schiff, 2008; Seth et al., 2005; Min, 2010; Edelman et al., 2011). In the anamniotes, such a reticular-thalamocortical complex has been proposed but never verified experimentally (Butler, 2008b). If present, it must be smaller, given the small size of the cerebral-cortex homologue (dorsal pallium) of lampreys and most other anamniotes (Murakami et al., 2001; Muhlenbrock-Lenter et al., 2009; Wullimann and Vernier, 2009b).

Some theories of primary consciousness, particularly those of Edelman (1989, 1992; Edelman et al., 2011), implicate memory functions as a key component. Memory construction in vertebrates is performed by the hippocampus (Martin et al., 2000; Jacobs, 2012). Although functional investigations are lacking for lampreys, their telencephalon does possess a hippocampus or medial pallium (Nieuwenhuys, 1977; Polenova and Vesselkin, 1993; Northcutt and Wicht, 1997; Nieuwenhuys and Nicholson, 1998). The lamprey hippocampus (PHip, see Figure 7) has the typical, widespread, connections within the brain, including to the dorsal thalamus, the optic tectum, dorsal pallium, and the olfactory bulb.

The neurohierarchical pathways for all major senses have been documented in lampreys, as summarized by Nieuwenhuys and Nicholson (1998). They are widely recognized to be very similar to those of other vertebrates, especially of other anamniotes (Binder et al., 2009).

In summary, the lamprey brain possesses all the requisite regions and neurohierarchical pathways for visual, olfactory, somatosensory and other images, integrated together to produce attention, awareness, neural synchronization and memory—all the elements proposed to be necessary for conscious awareness. Thus, given our assumptions of the sufficient neural underpinnings, we conclude that the lamprey has *at a minimum* sensory consciousness.

## Genes and consciousness

Consciousness may now be understandable from an entirely new perspective, that of genetics. Murakami and Kuratani (2008) found developmental-genetic evidence for our proposal that somatotopy and consciousness emerged during the transition from cephalochordate-like ancestors to vertebrates. They found in lampreys that the trigeminal sensory neurons project somatotopically to the relay nuclei in the hindbrain, with the neurons of these brain nuclei being organized in the same somatotopic pattern as in the trigeminal nerve. This somatotopic relationship also occurs in all gnathostome vertebrates, where the connections and nuclei are patterned by expression of Hoxa2, a genetic-transcription factor that also patterns the developing hindbrain into bulged segments called rhombomeres. *Significantly, Murakami and Kuratani demonstrated this same association of somatotopy, Hoxa2, and rhombomeres in the lamprey*. Thus, they tied one key component of consciousness, isomorphic somatotopy, to a specific gene, *Hoxa2*.

Second, *Hox*-gene expression in the rhombomeres, including expression of *Hoxa2*, is known to signal the patterning of neural crest in the vertebrate head (Trainor and Krumlauf, 2000), including signaling the crest-derived sensory neurons that are unique to vertebrates and are located at the start of the conscious-sensory pathway. In fact, the extensive gene networks involved in the development of neural crest and ectodermal placodes have been worked out in considerable detail (networks with *Snail1/2*, *FoxD3*, *Twist*, *Six1*, *Six4*, *Eya*, and many more genes; Sauka-Spengler and Bronner-Fraser, 2008; Schlosser, 2008; Shiau et al., 2008; Yu, 2010; Grocott et al., 2012). Thus, a second aspect of consciousness as we have proposed it, namely the neural crest- and placode-derived neurostructure, is tied to multiple, specific genes.

Third, genetic signals have been identified for the development of retinotopy in the brain (En-1 and En-2 signals: Friedman and O'Leary, 1996; EphrinA signals: Gosse et al., 2008; Bevins et al., 2011), as have genes in the ear related to tonotopy (*Tnc*, *Nov*, *Slo*: Frucht et al., 2011; Son et al., 2012). These pathways have been found in zebrafish, birds, and mammals, but have not been investigated in lampreys. Still, they show retinotopy and tonotopy to be two more aspects of proposed consciousness that are tied to specific genes.

In embryonic vertebrates, the dorsal pallium (likely critical for consciousness in mammals and birds) is characterized by expression of the Emx transcription factor. Emx is also expressed by the dorsal pallium of lamprey embryos (Murakami et al., 2001), although we reiterate that, except for olfaction, this region is not involved with isomorphic sensory consciousness in anamniotes (Wilczynski, 2009). Nonetheless, the fact remains that *all* groups of vertebrates express the *Emx* gene that has been associated with consciousness in mammals and birds; this ties another gene to consciousness. As for the optic tectum, which we associate with consciousness in anamniotes, key genes that signal its development are also known (*Otx, Fgf8, En*, and others: Friedman and O'Leary, 1996; Butler, 2000; Murakami et al., 2001; Rhinn et al., 2009; Sprecher, 2009).

By contrast, the non-vertebrate amphioxus has none of the structures that we associate with consciousness in vertebrates. It has no crest/placode-derived trigeminal or spinal sensory neurons, no Hoxa2-specified rhombomere segments (although it does express *Hox*2 in an unsegmented strip in its hindbrain), and it has no tectum, tectum genes, or telencephalic pallium (Wicht and Lacalli, 2005; Murakami and Kuratani, 2008; Schlosser, 2008; Yu, 2010; Pani et al., 2012; Figure 2A).

In summary, the genetic data support the existence of a hierarchy of somatotopy in lampreys that is based on the same gene suite as in mammals and other gnathostomes, and which evolved after the divergence from amphioxus (see the timeline in Figure 4 of Murakami and Kuratani, 2008). Thus, these genes could be a proxy for the appearance of consciousness at the start of the vertebrate line, the same timing we proposed based on other lines of evidence. Additional genes, which we associated with the retinotopic and tonotopic aspects of consciousness in vertebrates, also support this conclusion. Many of the genes are fully characterized down to their DNA sequences in multiple groups of vertebrates (e.g., the *Hox* genes: Takio et al., 2004). By our hypothesis, consciousness is in our genes, and some of these genes have been identified.

Table 2 lists all the neural features we have associated with consciousness in vertebrates, with genes that contribute to these features. It includes not only the genes we considered here in the text but also some other genes that are expressed in the receptors at the start of the sensory pathways.

## Discussion

### Recap and evolutionary shifts in sensory consciousness

Using multi-level, isomorphic sensory representations in vertebrates as a “marker” for the presence of sensory images and hence phenomenal, primary consciousness, the minimum requirement for such consciousness in chordates is a tripartite brain including a craniate forebrain (but not necessarily a highly developed dorsal pallium), a midbrain, and a hindbrain. We reasoned that this brain must feature: (1) a hierarchical system of isomorphically organized, reciprocally communicating, sensory-integration nuclei and centers, with conscious images emerging from the higher-level processing of different sensory modalities and submodalities (Table 1); and (2) interactions among the RAS, thalamus, and tectum or pallium that integrate sensory representations into a wide neural network that contributes to arousal and thereby to consciousness (Baars, 1988, 2002; Edelman, 1989, 1992; Newman and Baars, 1993; Crick, 1994; Llinas and Ribary, 2001; Ribary, 2005; Seth et al., 2005; Min, 2010; Edelman et al., 2011). By this reasoning, the cephalochordates and tunicates are not conscious because they lack the key features. But the lamprey, representing the most basal of the living vertebrates, has all these features and thus is hypothesized to possess sensory consciousness.

We suggest that during vertebrate history, the neural center of primary consciousness changed in two major steps. Multimodal and isomorphic sensory consciousness first evolved around the visual sense and thus initially centered in the visual tectum of the midbrain, although olfactory perception involved the telencephalic pallium (step 1: fish and amphibians). Direct evidence of eyes and indirect evidence of a tripartate brain, neural crest, and placodes occur in fossils from the Early Cambrian. From this we deduce *that sensory consciousness arose at least 520 million years ago*, and was a primary driver of vertebrate evolution because its participation is required for complex animals to exploit novel habitats by more effectively sensing the environment. Table 3 provides an evolutionary timeline for the emergence of consciousness in this first step.

**Table 3:** **Timeline of the emergence of critical features of sensory consciousness in vertebrates**.

**Feature/Taxon**	**Timeframe^1^ (million years ago)**	**Earliest fossil evidence (animal fossils)**	**References**
Ediacaran period	635–541	Sponges	Erwin et al., 2011; Erwin and Valentine, 2013
Cambrian period	541–488	Worm trace: *Treptichnus pedum*	Peterson et al., 2008; Erwin and Valentine, 2013
1. First chordate	560–520	No direct fossil evidence	See Figure 1, based on earliest body fossils of any bilaterian animals being about 560 my old (Erwin and Valentine, 2013)
2. Common ancestor of tunicates and vertebrates (and first precursors of placodes?)	560–520	*Shankouclava* (fossil tunicate)	See Figure 1; Chen et al., 2003; Sestak et al., 2013
3. Paired lateral eyes	560–520	*Haikouichthys*	Shu et al., 1999, 2003, 2009; Butler, 2000; Vopalensky et al., 2012
4. Fully differentiated tri-partite brain^2^	560–520 (460)	*Haikouella*, or the first gnathostome-fish fossils^2^	Janvier, 1996; Butler, 2000, 2006; Mallatt and Chen, 2003; Murakami and Kuratani, 2008; Sprecher, 2009
4. Cephalate animal	560–520	Hypothetical, so no fossil evidence	Butler, 2000, 2006
5. Placodes and neural crest	560–520	*Haikouella* and *Haikouichthyes*	Shu et al., 1999, 2003, 2009; Mallatt and Chen, 2003; Hall and Gillis, 2013
5. Isomorphic neural representations^2^	560–520 (460)		Murakami and Kuratani, 2008; Stephenson-Jones, 2012
5. Non-visual sense organs-1 (olfaction, trigeminal somatosensory)	560–520	*Haikouichthys* (and *Haikouella*?)	Mallatt and Chen, 2003; Shu, 2003; Shu et al., 2003
5. Sister group of vertebrates: *Haikouella*	520	Yunnanozoans	Chen et al., 1999; Chen, 2012
6. Non-visual sense organs-2 (equilibrium, taste? lateral line?)^2^	560–520 (460)	*Haikouichthys*, *Astraspis*, and *Sacabambaspis*	Sansom et al., 1997; Braun and Northcutt, 1998; Shu, 2003; Shu et al., 2003
7. Vertebrate: *Haikouichthys*	520	*Haikouichthys* and related genera	Shu et al., 1999, 2003, 2009

*Note the near-simultaneity of appearance of all features, near the time of vertebrate origin*.

*Features and taxa are numbered as 1–7 in the estimated order of their evolutionary appearance. Repeated numbers mean the different events occurred together or nearly simultaneously*.

1 *Dates of the features are taken from the fossil record (see text)*.

2 *These features are not directly observable in the 520 million-year-old Haikouichthys fossils, but are inferred to have existed in that vertebrate because some correlated structures did. Conceivably, the feature might date to as late as the cyclostome (agnathan)-gnathostome split at 460 mya (Mallatt, 1996), but no later because both lampreys and gnathostomes have it*.

Next, in a second major step, in ancient amniotes of the pre-mammal and sauropsid-reptile lineages, the dorsal pallium gradually became the dominant center of sensory consciousness, mostly independently in the two lineages (step 2: amniotes). The mammalian step culminated when true mammals evolved from mammal-like reptiles in the late Triassic (about 200 mya), and the sauropsid step somewhat later, in the first birds (around 180 mya). Actually, the step was probably gradual in both these lines of amniotes, its full duration spanning the Late Paleozoic and Early Mesozoic from roughly 350–180 million years ago (Benton and Donoghue, 2007). In the earliest mammals, this step to pallial consciousness can be related to a shift from vision to olfaction as the dominant sense (Rowe et al., 2011), but no such sensory shift occurred in the evolution of birds, where olfaction even declined (Roper, 1999). This inconsistency makes the change to pallial consciousness in birds difficult to explain. Still, one can speculate for both birds and mammals that when the center of sensory consciousness shifted from the optic tectum to the markedly enlarging and increasingly complex dorsal pallium, it involved an expansion and enrichment of the conscious experience.

The second step in mammals merits further consideration. It nicely shows that although brains became more complex during the half-billion year saga of vertebrate evolution, not all of the sensory systems did. The visual system of the early, nocturnal, mammals was regressed compared to that of their diurnal, highly visual reptilian ancestors (Bowmaker, 1998; Jacobs, 2009; Hall et al., 2012), and the proto-mammalian olfactory system was highly developed for a keen sense of smell (Rowe et al., 2011). Regression in vision probably explains why the tectum of mammals (superior colliculus) is less elaborate than that of extant reptiles and birds, who retained acute vision throughout their entire history (Aboitiz, 1992). When most orders of early mammals became diurnal again, probably after the extinction of the dinosaurs, vision became more important and the retina and visual areas of the cerebral cortex expanded in size and complexity. This especially occurred in the keen-sighted primates, where the regions for olfactory processing were reduced (Allman, 1999). With these back-and-forth shifts in the dominant sense during mammalian evolution, the central hub of sensory-conscious experience shifted between the olfactory and visual cortex.

Additionally, different sensory systems became highly elaborate or regressed in other lines of vertebrates. One example is the extreme development of electroreception in some teleosts (“electric fish”) with enlargement of the processing part of their cerebellum and cerebral pallium (Prechtl et al., 1998; Wullimann and Vernier, 2009a). Another, more dramatic example is the sister cyclostome of the lamprey, the hagfish, whose nervous system and proposed consciousness were shaped by their unusual lifestyle of burrowing in soft sediment of the dark ocean floor (Mallatt, 1997). Hagfish have a regressed visual system, a rudimentary lateral line, and perhaps a simplified inner ear, but they enjoy highly sensitive olfaction, exaggerated touch perception, and abundant taste-like chemoreceptors in the skin of their head (Andres, 1993; Braun, 1996; Braun and Northcutt, 1998; Von During and Andres, 1998; Lamb, 2013), all of which help them to locate and feed upon carcasses on the ocean bottom and to prey on live worms and burrowed fish within the sediment (Zintzen et al., 2011). Along with these “extreme senses,” the hagfish has a large, well-developed brain (Wicht, 1996; Ronan and Northcutt, 1998; Wicht and Nieuwenhuys, 1998; Wicht and Northcutt, 1998). This brain features all the major sensorimotor systems with the exception of an oculomotor system, and has especially robust olfactory and trigeminal-sensory representations. What is it like to be a hagfish? Any conscious experience would center on three-dimensional mental “images” of richly perceived and spatially discriminated smells, touch sensations, and taste stimuli, all in virtual blindness.

### Objections to the hypothesis

#### Challenge 1: isomorphism does not equal consciousness

Our thesis could be challenged in four ways. First, one might argue that isomorphic representation cannot be equated with consciousness because artificial sensors and computers can receive and map out stimuli, yet these machines are not conscious. In response, we reiterate that our hypothesis states that sensory consciousness and isomorphic representations entail a highly specific “*kind”* of isomorphic representation, not just *any kind*. The brain possesses an entirely *unique* architecture that features—in addition to a huge “computer-like” amount of complex processing—reciprocal communication between the levels of the neural hierarchy with integrated and novel emergent properties appearing with the addition of each level. Thus, the neural hierarchy represents a unique neurobiological substrate and organization quite different from that found in computers made of silicon chips and wires (Feinberg, 2012).

#### Challenge 2: consciousness is “corticothalamic” and should be studied from the top down

The second challenge says it is better to search for non-human consciousness by starting with entities known to be conscious. That is, begin with humans and the animals most closely related to us, namely apes and the other mammals and then search carefully, using homology and analogy, for signs of consciousness in slightly simpler brains such as those of reptiles. Most studies of comparative animal-consciousness proceed this way (e.g., Edelman et al., 2005; Butler, 2008a; Mashour and Alkire, 2013). This is preferable, it could be said, to our seeking consciousness in the distantly related lower-vertebrates like lampreys, where any version of consciousness could be strange or absent and therefore harder to recognize, to prove, or to disprove. The human-first approach is top-down, whereas ours is bottom up.

A potential weakness of our bottom-up approach—searching for consciousness in the simplest animals that may have it—is that this approach requires foreknowledge of and a consensus on the *minimally sufficient neural underpinnings for consciousness*, and this is lacking. However, the bottom-up approach is still worth exploring if a well-specified and plausible hypothesis about these underpinnings can be provided. That is what we attempt to provide with our hypothesis that sensory consciousness emerges from the tripartite brain, isomorphic representations in neural hierarchies, and the attention-directing feature. We also re-emphasize that our bottom-up hypothesis is not claiming fish and amphibians have a full-blown, human-like, self-reflective consciousness (Boly and Seth, 2012), only that they experience in-the-moment ‘sensory mental images’ or qualia, which is all that is required for the existence of sensory consciousness (Revonsuo, 2006).

An important basis of the human-centered, top-down approach is that in humans and other mammals, much evidence attributes consciousness to the large cerebral cortex and to its interactions with the thalamus. More specifically, the many widely distributed areas of the cortex have reciprocal (reentrant) patterns of synchronized communication with one another and with the thalamus while the RAS-related “central nuclei of the thalamus” subserve arousal and attention (Schiff, 2008; Edelman et al., 2011). In humans, damage to this thalamocortical complex causes disturbances in consciousness (Schiff et al., 2007; Boly and Seth, 2012), and there is little evidence for conscious content in any other regions of the mammalian brain, such as the brainstem. In birds, similarly, conscious functions are being identified in their cerebral-cortex homologues, the Wulst and the dorsal ventricular ridge (DVR) (Rose et al., 2009; Dugas-Ford et al., 2012; Karten, 2013). Overall, this idea of the “exclusivity” of the cerebral cortex and thalamocortical complex in consciousness is a version of a “corticothalamic hypothesis” of mammalian consciousness, a good summary of which is provided by Edelman et al. (2011). A sample of the many other studies that follow or support it includes Llinas and Ribary (2001); Baars (2002); Butler et al. (2005); Ribary (2005); Seth et al. (2005); Butler and Cotterill (2006); Steriade (2006); Butler (2008a); Min (2010); van Gaal and Lamme (2012), and Baars et al. (2013).

In our view the corticothalamic hypotheses, *if applied to all vertebrates*, underestimate the potential role of the optic tectum in consciousness, specifically in fish and amphibians. That is, these hypotheses miss the tectum's participation in the potentially conscious functions of recognizing and perceiving objects and directing attention to important objects in the visual field (Dicke and Roth, 2009; Saidel, 2009). Sophisticated visual functions are also performed by the large tecta of *birds* (Shimizu et al., 2009; Wylie et al., 2009). This raises the possibility that avian visual consciousness is shared by their tectum and their visual telencephalon (Wulst). Reptiles also have a large optic tectum, a large DVR, and a cerebral cortex (albeit simple in nature), suggesting to us that the tectum and dorsal pallium also share the conscious functions in reptiles. Thus, corticothalamic hypotheses may not apply fully to reptiles or birds.

We have proposed that the brain's main center of sensory consciousness shifted from the tectum to the telencephalic pallium around 350–180 mya, independently in the mammalian and sauropsid lines. Ironically, corticothalamic hypotheses of consciousness (Butler et al., 2005; Edelman et al., 2005) also view this Paleozoic/Mesozoic timeframe as pivotal, but instead assign to it the original *appearance* of vertebrate consciousness in the sauropsid/bird and mammal lines.

Another “corticothalamic” argument could be raised, against our claim that sensory consciousness in anamniotes is tectal not telencephalic. This argument points out that in certain fish lineages the sensory *telencephalon* is also large and elaborate (Huesa et al., 2009; Wullimann and Vernier, 2009a,b,c), raising the possibility of extensive pallial participation in the consciousness of these anamniotes. In some sharks, the dorsal pallium is expanded with higher-order sensory-processing nuclei. The same is true in some teleosts (Prechtl et al., 1998), although within a uniquely everted pallium that indicates an independent evolution of this expansion in the teleost line (Wullimann and Vernier, 2009b). On the other hand, the dorsal pallium of many other anamniotes is small and that of lampreys is tiny, so our idea that sensory consciousness in vertebrates was initially tectum-centered still holds. However, we must admit that we may have underestimated the *potential* for an increasing role of the dorsal pallium in the sensory consciousness of all the large-telencephalon vertebrates, including those that are fish.

The corticothalamic hypotheses have difficulties of their own. Because they are top-down, constructing and testing them means starting with the staggering complexity of mammalian brains. By contrast, our bottom-up approach starts with more-tractable objects of study, namely isomorphic neural hierarchies and the least complex of all vertebrate brains (that of the lamprey), while still avoiding potentially over-simplified and neurally remote approaches to consciousness that are based on synthetic neural modeling with computers, equations, artificial intelligence, and robots (Reeke and Sporns, 1993; Gale et al., 2001; Franklin, 2005; Tonini, 2008; Nageswaran et al., 2009; Ramamurthy and Franklin, 2009).

The corticothalamic approach, as it has been used to date, studies only humans and other higher vertebrates in order to draw conclusions about consciousness in lower vertebrates. This risks looking in exactly the wrong place by ignoring the only relevant study animals. *It seems much better to seek anamniote unconsciousness and sensory consciousness directly in the fish and amphibians, which themselves are readily available for experimental study*.

#### Challenge 3: unconscious hierarchies

The third challenge says our hypothesis is invalid if its marker for consciousness, complex hierarchies of isomorphic representations, exists for any *unconscious* sensory processing. Actually, our hypothesis does not claim that *all* such isomorphic hierarchies *must* be conscious. We recognize that the lower levels of some isomorphic sensory hierarchies can influence behaviors without involving consciousness (e.g., vestibular reflexes; Kandel et al., 2012). We also recognize that isomorphic sensory systems have some major aspects that are conscious and others that are non-conscious (e.g., visual recognition of fearful or fear-expressing faces, and the just-mentioned vestibular system; Williams et al., 2004; Chen et al., 2010). And it is certainly true that some lower-order sensory systems are not consciously perceived at all (e.g., tendon stretch, the visceral-sensory paths from taste-like chemoreceptors in the lining of our respiratory tubes, and baroreceptors in the carotid sinuses; Mescher, 2010; Kandel et al., 2012). Additionally, many aspects of proprioception, although isomorphically represented, are unconscious. Merker (2005) suggests such unconscious proprioception is necessary because too much sensory information about bodily movements would confound and contaminate the “stable reality space” of our consciousness, thus interfering with its key role in guiding goal-directed actions. But on the whole, we feel that consciousness uses, rather than excludes, the important classes of sensory information. That is, primary consciousness mainly involves the senses most vital for survival. The intricate, consciously perceived, sensory maps of an animal's outer world (and inner world) lead to improved information-gathering and better decisions on how to respond to complex and changing environments (Plotnick et al., 2010).

#### Challenge 4: unconscious perception

Numerous types of unconscious (=non-conscious) perception have been identified in humans and could be used to argue that lower vertebrates only have such unconscious perception and are not conscious. Such unconscious processes, extensively investigated, include blindsight, subliminal perception during masking, binocular rivalry, attentional blink, inattentional pre-consciousness, and implicit cognition (Baars, 2002; Kouider and Dehaene, 2007; Ciaramelli et al., 2010; Brogaard, 2011; Overgaard, 2012; Panagiotaropoulos et al., 2012; van Gaal and Lamme, 2012; Baars et al., 2013). We will take these investigations at face value, ignoring the possibility that some of their “unconscious” stimuli were actually consciously perceived (“weakly glimpsed” or incompletely blocked: Overgaard et al., 2008, 2013). Even so, we find several reasons why unconscious perception in humans is mostly irrelevant to the question of consciousness in non-mammalian anamniotes. For instance, in humans, when compared with conscious vision, unconscious vision is limited in duration, in flexibility, and in the strategic use of its information for decision making (van Gaal et al., 2012), which would compromise the survival of any animal that relied on unconscious vision alone. Even those unconscious processes that are most likely to influence human behavior (mismatch negativity, response inhibition, error detection, conflict resolution: Naatanen et al., 2007; Kiefer et al., 2011; van Gaal and Lamme, 2012) are weaker than, or intimately tied to, or dependent upon, a *conscious* neural activity, which dominates the perception. It seems that human (cerebral) *un*consciousness is too intricately linked to human (cerebral) consciousness for it to be relevant to the *non-cerebral* kind of consciousness we propose for fish and amphibians.

Perhaps the best illustration of the above considerations is the phenomenon of blindsight. This results from damage to the primary visual cerebral cortex, or V1, in the occipital lobe of humans. With the affected individuals lacking primary central visual-pathways, in the vast majority of cases their visual function is severely degraded and functionally useless, and whatever residual, non-conscious visual functions are preserved are highly degraded (Alexander and Cowey, 2010; Cowey, 2010; Overgaard, 2012).

Thus, a fish or frog with only blindsight or subliminal senses would have such limited awareness as to be fast prey; it could never survive. Even lampreys have complex behavioral adaptations, dependent on vision and other senses, that far exceed what could be produced by human paradigms of unconscious perception. Parasitic lampreys use vision, smell, and probably electroreception to track their prey fish, to avoid danger, locate mates, and court (Hardisty, 1979; Chung-Davidson et al., 2004). They migrate long distances and use odors, including pheromones, to find appropriate streams for spawning (Vrieze and Sorensen, 2001; Vrieze et al., 2010). Perhaps the pinnacle of such behavioral complexity is the sneak-mating strategy used by male lampreys in the spawning nest, in which a sneaker male lurks near a spawning pair, waits until the proper time, and the wraps its body around the pair to spew its own milt onto the female's newly released eggs (Hume et al., 2013). It seems to us that such intricate and varied survival functions require the heightened awareness of sensory consciousness.

However, the fact that humans cannot perform many normal functions without consciousness does not necessarily *prove* that these functions cannot be done without consciousness in more basal vertebrates or in non-neurobiological systems. For instance, many highly complex functions are done by computers without consciousness (*conscious inessentialism*; see Moody, 1994; Flanagan, 1997). However, if conscious inessentialism were true for the conscious functions of the human brain, then, theoretically, the normally conscious sensory functions could also be performed without consciousness. The problem for neurobiology is that this leaves unexplained, for example, why certain behavioral functions are lost in a human with a lesion in the isomorphic visual cortex V1, while other, non-conscious, functions are preserved. In the human, the lost functions require consciousness and the most parsimonious explanation for this seems to be that, indeed, the documented sensory isomorphism of all primary sensory areas of the cortex is necessary for sensory consciousness.

Nonetheless, is it still conceivable that fish, amphibians, and reptiles exclusively use elaborate yet “unconscious” processes in their advanced survival behaviors, processes that could be unlike and far beyond the non-adaptive unconscious processes that are typically studied in the above-mentioned human experiments. The reasoned assumption of our hypothesis—that certain isomorphic neural hierarchies are required for biological consciousness—could refute this challenge, but it does so only if the assumption is true. Thus, our assumption must be evaluated, by testing the predictions of our hypothesis in anamniotes, ideally by testing them in a way similar to how the rival, corticothalamic, hypotheses have been tested in mammals.

### Tests to meet the challenges

Our hypothesis that sensory consciousness depends on isomorphic hierarchies, and on the optic tectum in anamniotes, can be tested in two ways. One way is to record the electrophysiological properties of the tectum, thalamus, cerebral pallium, and reticular formation in fish and amphibians, expanding on the thalamocortical recordings reviewed by Llinas and Steriade (2006), but focusing on the thalamo-tectal interactions in these anamniotes. Reentrant and recurrent processing should be sought between thalamus and tectum, as should oscillatory synchronicity of the firing neurons. Recurrent processing (van Gaal and Lamme, 2012) means that whenever sensory information reaches a successive level in a hierarchy, the higher level sends signals back to the lower levels, as has especially been demonstrated in the cerebrum of higher primates. This recurrent interaction could be a hallmark of consciousness because it combines information from many different brain regions and its reverberations allow it to retain information over time. Forward-then-recurrent signals should be easy to detect with the recording electrodes. Large, sluggish frogs and young zebrafish (Ahrens et al., 2012) should be the best test animals for the brain recordings we propose.

Another test is to perform such recordings on *reptile* brains. Reptiles are the key transitional group for all the hypotheses of consciousness considered in this paper, yet they are exceptionally poorly studied with regard to sensory processing and sensory consciousness (Butler and Cotterill, 2006; Jarvis, 2009; Dugas-Ford et al., 2012).

### Different genes and different evolutionary trajectories

Can the genes we have related to consciousness tell anything about the origin and earliest evolution of the conscious process? One might predict that the developmental pathways involved in the different aspects of isomorphism share genes and gene circuits in common, at least in the fundamental, early expressed stages of these pathways, and that these shared genes should derive from the visual pathway if vision was the ancestral sense around which all other senses were patterned (Butler, 2000; Shepherd, 2012). But this prediction fails for several reasons. The gene-expression pathways in the different kinds of ectodermal placodes differ from one another (Graham and Shimeld, 2012)—although these differences can be explained as separate ways to direct the placodes away from the default, ancestral state of developing into the lens placode of the visual system (Bailey et al., 2006). This lens-as-template finding rescues the “vision-first” hypothesis, but then, comparisons of the all-important placodes with the neural crest proceed to reveal even more genetic disunity. For example the neural-crest specifiers (transcription factors Snail1/2, FoxD3, Twist) differ markedly from the placode specifiers (Six1, Six4, Eya) (Yu, 2010; Graham and Shimeld, 2012). In fact, Schlosser (2008) demonstrated in depth that the neural crest and placodes differ from each other not only in their specifier genes, but also in the upstream gene pathways that induce their formation in the first place, which led him to conclude the crest and placodes evolved independently of one another from the dorsal ectoderm (also supported by Sestak et al., 2013). And the “isomorphism” genes we listed in the “Genes and Consciousness” section as establishing trigeminal-somatotopy (*Hoxa2*), vs. retinotopy (*EphrinA, En-1, En-2*), vs. tonotopy (*Tnc*, *Nov*, *Slo*) all differ from each other. Thus, the modules of the isomorphic neural hierarchy show no genetic commonality, judging from the available data. This implies that when the definitive placodes and neural crest evolved as key adaptations in pre-vertebrates in the Early Cambrian, the genetic signals for patterning the different senses diverged rapidly into a multitude of different directions and pathways. It also implies that many aspects of isomorphic mapping in the nervous system evolved independently among the different senses.

Similarly, consciousness itself (as we have proposed it) appears to have advanced through somewhat different paths in different lines of vertebrates. One example is the tectal consciousness of anamniotes vs. the pallial consciousness of amniotes. Another, puzzling, example comes from comparing the pallial consciousness of birds vs. mammals. While birds are capable of many advanced cognitive abilities such as demonstrating working memory and episodic memory, learning, category formation, tool use, number concepts, and object permanence (Butler et al., 2005; Edelman et al., 2005; Butler, 2008a; Edelman and Seth, 2009), the pallial regions involved seem to differ from those of mammals that have these same abilities. That is, the bird's Wulst and DVR do not obviously resemble the mammal's cerebral cortex in gross anatomy nor in the microscopic anatomy that could be studied by traditional microscopic methods. This led to much controversy over the avian homologue of the mammalian cerebral cortex (Reiner, 2005; Medina, 2009; Butler et al., 2011).

New studies finally solved this puzzle (Wang et al., 2010; Kaas, 2011; Dugas-Ford et al., 2012; Karten, 2013), by confirming that the laminated cerebral cortex of mammals corresponds to both the non-laminated DVR and the pseudo-laminated Wulst of birds, as well as to the DVR and simpler cerebral cortex of reptiles. The studies succeeded by showing that in the three animal groups these brain regions have comparable gene-expression patterns (by EAG2, RORB, ER81, PCP4), as well as comparable afferent and efferent connections (also see Jarvis, 2009; Kalman, 2009; and Butler et al., 2011). In addition, the new studies revealed a nearly identical internal microcircuitry in birds and mammals. Finally and most importantly, the corresponding avian and mammalian regions are known to feature the same kinds of retinotopic, somatotopic, and tonotopic maps, as well as similar higher-order sensory processing centers (Adamo and King, 1967; Karten, 1968, 1969; Karten et al., 1973; Pettigrew and Konishi, 1976; Pettigrew, 1979; Wild, 1987; Wang et al., 2010). Thus, despite some divergent and parallel evolution of their homologous pallial regions, the large-brained mammals and birds should share ways of generating higher sensory consciousness with hierarchical organization and isomorphic maps.

## Conclusion

This is the first hypothesis that dates the origin of consciousness, explains its neural architecture, explores its genetics, identifies the most basal animal that has it, and accommodates its neurobiology with the “hard problem” of consciousness and subjectivity, all knitted together in one model. We hypothesize that primary or sensory consciousness stems from a confluence of neurological features common to all vertebrates (Table 2), especially from multiple, reciprocally connected, isomorphic representations at different hierarchical levels within the nervous system. These features co-emerged, probably in a rather short interval of time, between 560 and 520 mya (Table 3). Perhaps over half a billion years old, consciousness may be hundreds of millions of years older than investigators have supposed, so we cannot assign it exclusively to the animals with the highest intellectual capacities, such as humans, apes, and porpoises. We also hypothesize that from the start, sensory consciousness and the existence of qualia acted as a prime mover of vertebrate evolution by allowing vertebrates to go beyond mere reflexes and to map, then assess, their external and internal environments in exquisite detail.

We offer a “two step hypothesis” in which the neural center of primary consciousness first evolved in Cambrian anamniotes as multimodal and isomorphic sensory representations dependent on the visual tectum and its interconnections with the thalamus and reticular formation. Second, in reptiles of the pre-mammal and sauropsid lineages, the enlarging dorsal pallium gradually became the dominant center of sensory consciousness, mostly independently in the two lineages. The timing for this *second* step at 350–180 mya corresponds to the initial emergence of consciousness in rival, corticothalamic hypotheses (Butler et al., 2005; Edelman et al., 2005).

We urge that more research be performed directly on conscious and unconscious senses in anamniotes and reptiles, rather than inferring the mental states of such vertebrates based on the mammalian (and avian) pallium and thalamus, which may be poor models.

We further posit that there is a common genetic basis for consciousness, not shared among all the different senses, but shared within the individual sensory systems across the vertebrate groups (fish, amphibians, reptiles, birds, and mammals). Some of the important genes are now known (Table 2), and many more will undoubtedly be discovered. These genes may be useful markers for the presence of primary consciousness. Assuming that consciousness in *invertebrates* either does not exist or that it arose later (e.g., in cephalopods, see Kroger et al., 2011), then the most basal conscious organisms on earth are the jawless vertebrates, represented by the lamprey.

Finally, given the existence of isomorphic neurohierarchical processing in all vertebrates, the neuroontologically unique and irreducible features of sensory consciousness appear to require successive re-representations of mapped sensory information throughout the levels, coupled with a global activation system (Feinberg, 2009, 2011, 2012). The qualitative “feel” of these sensory images had its origin in sensory receptors that subsequently proceeded to evolve and specialize within the hierarchical pathways. In evolution, these systems began as simple, unconscious reflexes (e.g., the light spot-initiating reflexes in amphioxus, or nociceptors stimulating reflexive withdrawal), but as the central structures evolved to process this activity, hierarchically arranged *neural-neural interactions* created conscious sensory images and their associated qualia (Feinberg, 2012). This made sensory consciousness into the *unique neurobiological system feature* that it is.

### Conflict of interest statement

The authors declare that the research was conducted in the absence of any commercial or financial relationships that could be construed as a potential conflict of interest.
